# ROS-Responsive Double-Layer Microneedles Enable Sequential Antibacterial and Immunomodulatory Therapy for Infected Wound Healing

**DOI:** 10.7150/thno.122865

**Published:** 2026-01-01

**Authors:** Huifang Zhang, Zhongke Wang, Yujing Zhu, Ting Huang, Ziliang Xiu, Haozhe Huang, Hankai Li, Jing Xie, Haixia Huang, Min Liu, Libo Sun, Yuyan Lan, Ling Guo

**Affiliations:** 1 Oral & Maxillofacial Reconstruction and Regeneration of Luzhou Key Laboratory, The Affiliated Stomatological Hospital, Southwest Medical University, Luzhou 646000, China.; 2 Department of Prosthodontics, The Affiliated Stomatological Hospital of Southwest Medical University, Luzhou 646000, Sichuan Province, China.; 3 Institute of Stomatology, Southwest Medical University, Luzhou 646000, China.; 4 Department of Stomatology, Sichuan Provincial People's Hospital, School of Medicine, University of Electronic Science and Technology of China, Chengdu 611731, China.; 5 Stomatological Hospital of Chongqing Medical University, No. 426 Songshibei Road, Yubei District, Chongqing 401147, China.; 6 Department of Stomatology, Luzhou People's Hospital, Luzhou 646000, China.

**Keywords:** Microneedles, Infected wound healing, ROS-responsive, Antibacterial, Immunomodulatory

## Abstract

**Background:** The healing of chronically infected wounds is severely hindered by persistent inflammation, bacterial infection, and oxidative stress, posing great challenges to clinical therapy. To address these challenges, we designed a multifunctional dual-layer microneedles patch (MN@DOX+RES) featuring reactive oxygen species (ROS) responsiveness and dual drug delivery capabilities. This patch is engineered to deliver synergistic antibacterial, anti-inflammatory, and antioxidant effects, thereby promoting the healing of infected wounds.

**Methods:** The dual-layer microneedles patch comprises a rapidly dissolvable HA backing layer loaded with DOX and a ROS-responsive tips layer composed of a crosslinked AHA-PBA/PVA matrix that encapsulates water-soluble RES inclusion complexes. A series of *in vitro* experiments was conducted to evaluate the mechanical strength, biocompatibility, antibacterial activity against *Staphylococcus aureus* and *Escherichia coli*, antioxidant performance, and macrophage polarization. *In vivo* evaluations were performed on rat models with infected skin wounds.

**Results:** The MN@DOX+RES microneedles exhibited strong skin penetration ability and excellent mechanical strength. It significantly inhibited bacterial growth, efficiently scavenged free radicals, reduced intracellular ROS levels, and enhanced M2 macrophage polarization. *In vivo*, the patch accelerated wound closure, suppressed the inflammatory cytokine IL-6, enhanced IL-10 expression, and activated the Keap1/Nrf2/HO-1 antioxidant signaling pathway.

**Conclusions:** This study proposes an innovative therapeutic strategy that combines dual-drug delivery, oxidative microenvironment regulation, and immune modulation to promote the healing of chronic infected wounds. The MN@DOX+RES microneedles system demonstrates great potential in overcoming clinical challenges associated with infection, inflammation, and the limitations of conventional therapeutic approaches.

## Introduction

The skin serves as the primary barrier that protects the human body from external environmental threats 1. However, its integrity can be compromised by both intrinsic (e.g., diabetes) and extrinsic factors (e.g., surgical procedures, trauma, and burns), leading to wound formation. These breaches in skin continuity provide entry points for pathogens such as bacteria, fungi, and viruses 2,3. The physiological wound healing process consists of four overlapping phases: hemostasis, inflammation, proliferation, and remodeling 4. Among the challenges in this process, bacterial infection poses a great risk 5. Invading bacteria colonize the wound site early on the surface and form biofilms by secreting extracellular polymeric substances 6. These biofilms, typically 100-2100 μm thick, act as a physical barrier that limits the penetration of therapeutic agents 7. The persistent presence of bacteria exacerbates the inflammatory response by promoting M1 macrophage polarization and excessive production of pro-inflammatory cytokines and proteases 8,9. Concurrently, elevated oxidative stress levels in inflammatory cells deep within the wound will lead to excessive production of reactive oxygen species (ROS) 10. When ROS accumulation exceeds physiological levels, it not only prolongs inflammation but also causes detrimental effects such as impaired collagen deposition and disrupted angiogenesis 11,12. These pathological changes collectively hinder wound healing and significantly reduce the patients' quality of life. Therefore, optimizing the wound microenvironment through effective antibacterial action, inflammation mitigation, and oxidative damage reduction is critical for accelerating healing.

In recent years, various topical wound dressings have been developed to enhance the healing process, particularly through localized drug delivery strategies 13,14. Among them, microneedles (MN), as a new type of transdermal drug delivery tool, have attracted much attention due to their advantages of being painless, minimally invasive, and capable of local drug delivery 15. Based on differences in structural design and mechanism of action, MN technology encompasses multiple types, such as solid MN, hollow MN, coated MN, dissolving MN, and hydrogel-forming microneedles (HFM) 16,17. Among these, HFM exhibits excellent biocompatibility and can be completely absorbed by the skin, eliminating concerns about microneedle residue and biohazardous waste 18,19. HFM can be composed of swellable polymers, which form a hydrogel network through crosslinking between polymer chains, facilitating high-dose drug loading and release 20.

Despite these advantages, HFM faces significant challenges in practical applications. Wound healing is a multifactorial, multistage, progressive process, demanding temporally and spatially controlled delivery of specific therapeutics at distinct phases 21. Crucially, the wound microenvironment undergoes dramatic shifts, particularly in the levels of ROS, which surge during the inflammatory phase but need resolution for progression to proliferation and remodeling 22,23. However, conventional single-layer HFM systems typically provide static or pre-programmed release profiles, which are unable to dynamically sense and respond to these evolving biochemical cues within the wound bed 24. This lack of responsiveness and inability to strategically compartmentalize drugs for targeted delivery to different wound depths or phases significantly hinders their efficacy in complex, dynamic healing scenarios. Therefore, there is a compelling need to develop next-generation HFM that incorporate ROS-responsive elements and spatially stratified architectures (e.g., bilayer designs) to achieve intelligent, stage-adaptive therapy.

To address the multifaceted challenges of infected wound healing, specifically combating infection, quenching excessive oxidative stress, and modulating dysregulated inflammation, a rational combination therapy is essential 25,26. In this study, we strategically selected two potent therapeutic agents: resveratrol (RES) and doxycycline (DOX). DOX, as a broad-spectrum antibiotic, possesses potent antibacterial activity and is crucial for eliminating pathogenic bacteria on wound surfaces and preventing biofilm formation 27. Concurrently, RES, a natural polyphenol, offers powerful antioxidant and anti-inflammatory properties. It effectively scavenges deleterious ROS, mitigates oxidative damage to cells and tissues, and helps reduce excessive inflammatory cascades deep within the wound 28-30. Critically, these two agents exhibit synergistic potential: DOX rapidly controls infection, reducing the bacterial load and associated inflammatory stimuli, while RES counteracts the infection-induced and inflammation-driven oxidative burst and promotes resolution of inflammation. This complementary action targets the interconnected pathological triad of infection, inflammation, and oxidative stress, collectively fostering a more conducive microenvironment for healing.

Building upon this therapeutic rationale, we engineered a dual-layer MN patch, designated MN@DOX+RES, which enables spatially and temporally controlled drug release in response to the wound microenvironment. The tips layer consists of a ROS-responsive hydrogel, synthesized *via* dynamic boronic ester bonds between 3-aminophenylboronic acid-grafted aldehyde-modified hyaluronic acid (abbreviated as AHA-PBA) and polyvinyl alcohol (PVA). Upon insertion into the wound, the hydrogel swells and anchors *in situ*. Elevated ROS levels at inflamed sites trigger bond cleavage, enabling localized and on-demand release of RES. The backing layer, composed of hyaluronic acid (HA) incorporating DOX, rapidly dissolves upon contact with wound exudate, ensuring fast and effective antibacterial action at the wound surface. This stratified design allows RES to act responsively within deeper tissues to alleviate oxidative stress and inflammation, while DOX acts rapidly at the surface to control infection. In this study, we performed a comprehensive evaluation of the morphology, mechanical properties, skin penetration, and drug release characteristics of the dual-layer MN@DOX+RES patch. *In vitro* and *in vivo* evaluations demonstrated excellent biocompatibility, potent antibacterial activity, significant ROS-scavenging capability, and strong anti-inflammatory effects. In a rat model of infected skin wounds, the patch significantly accelerated healing compared to both untreated controls and single-drug-loaded MN. Mechanistic investigations revealed that MN@DOX+RES activated the Keap1/Nrf2/HO-1 signaling pathway, enhancing endogenous antioxidant defenses and contributing to inflammation resolution. These findings highlight MN@DOX+RES as a smart, multifunctional wound dressing that combines physical protection, fast antibacterial activity, ROS-triggered therapeutic release, and activation of cytoprotective pathways, thereby offering a promising strategy for the treatment of complex infected wounds.

## Materials and Methods

### Materials

Hyaluronic acid (HA), polyvinyl alcohol (PVA), Sodium periodate, ethylene glycol, potassium bromide, 3-aminophenylboric acid (3-APBA), resveratrol, hydroxypropyl-β-cyclodextrin (HP-β-CD), Hydrogen Peroxide (H_2_O_2_) Content Assay Kit, Oxygen Free Radical (·O₂⁻) Content Assay Kit, and Hydroxyl Radical (·OH) Scavenging Capacity Assay Kit were purchased from Macklin Biochemical Co., Ltd (Shanghai, China). Doxycycline hydrochloride (DOX), 2,2-diphenyl-1-(2,4,6-trinitrophenyl) hydrazide (DPPH), 2,2′-azino-bis (3-ethylbenzthiazoline-6-sulfonic acid) (ABTS) and phosphate-buffered saline (PBS, 1X) were purchased from Solarbio Life Sciences Co., Ltd (Beijing, China). Dulbecco's Modified Eagle Medium (DMEM), 0.25% Trypsin-EDTA, penicillin-streptomycin, the Calcein-AM/Propidium iodide (PI) Live/Dead Viability/Cytotoxicity Assay Kit, 2',7'-dichlorofluorescein diacetate (DCFH-DA) were purchased from Beyotime Biotechnology (Shanghai, China). Fetal Bovine Serum (FBS) was purchased from PAN Biotech UK Ltd. Live/Dead bacterial staining kits were obtained from Bestbio Biotech (Shanghai, China). All other materials and reagents for this study were applied directly without further purification.

### Preparation and Characterization of the AHA-PBA/PVA Hydrogels

#### Synthesis and Formation of AHA-PBA/PVA Hydrogels

The fabrication process consisted of three key steps: the synthesis of aldehyde-modified hyaluronic acid (AHA), the preparation of 3-aminophenylboronic acid-grafted AHA (AHA-PBA), and the formation of AHA-PBA/polyvinyl alcohol (PVA) hydrogels (AHA-PBA/PVA hydrogels) *via* dynamic boronate ester crosslinking.

First, to synthesize AHA, HA (1.0 g) and sodium periodate (NaIO_4_, 0.5 g) were dissolved in 100 mL of PBS and stirred for 5 h at room temperature in the dark. The reaction was quenched with ethylene glycol (1 mL), followed by dialysis against deionized water for 3 days. The dialyzed product was lyophilized to obtain AHA. Subsequently, AHA-PBA was synthesized *via* a Schiff base reaction by dissolving AHA (1.0 g) and 3-APBA (2.5 mmol) in 100 mL of PBS and stirring the mixture for 24 h at room temperature. The solution was then dialyzed and lyophilized. Finally, to form the AHA-PBA/PVA hydrogel, AHA-PBA and PVA were individually dissolved in PBS at 5% (w/v). The two solutions were mixed at a 3:1 (v/v) ratio and briefly shaken to initiate gelation *via* dynamic boronate ester crosslinking.

#### Characterization of AHA-PBA/PVA hydrogels

The microstructure of the freeze-dried AHA-PBA/PVA hydrogels was observed using scanning electron microscopy (SEM, JEOL FESEM6700F). Ultraviolet-visible (UV-vis) spectroscopy (TU-1810, Beijing, China) was used to record the absorbance spectra (190-500 nm) of HA, AHA-PBA, and 3-APBA solutions at 1% (w/v). Fourier-transform infrared (FTIR) spectroscopy (WQF-530, Beijing, China) was conducted in the 4000-400 cm^-1^ range to confirm the functional groups present in HA, AHA-PBA, and 3-APBA. Nuclear magnetic resonance hydrogen spectroscopy (^1^H NMR) spectroscopy (500 MHz, Bruker) was employed to analyze the chemical structures of HA and AHA-PBA, and the degree of boronic acid substitution was calculated by comparing the integral areas of aromatic protons (7.5-8.0 ppm) and methyl protons (2.0 ppm). Additionally, rheological behavior was evaluated using a Kinexus Pro rheometer (Malvern) with a 10 mm parallel plate geometry. Shear viscosity (0.1-100 s^-1^) was recorded to assess the flow characteristics under increasing shear rates, and strain sweep (0.1-1000%) and frequency sweep (0.1-10 Hz) tests were conducted to assess the viscoelastic properties of the hydrogel.

To evaluate the degradation behavior, 400 μL of preformed AHA-PBA/PVA hydrogel was placed in centrifuge tubes and immersed in 1 mL of PBS containing 0, 100, and 500 μM H_2_O_2_. The total mass of each tube was recorded at the beginning, and the remaining mass was measured daily after carefully removing the supernatant. Fresh H_2_O_2_-containing PBS was replenished daily, and the degradation process was monitored until the hydrogel was completely degraded.

### Preparation and Characterization of RES Inclusion Complexes

#### Preparation of RES Inclusion Complexes

The RES inclusion complexes were prepared following the protocol described in the literature 31. Specifically, 1.54 g of hydroxypropyl-β-cyclodextrin (HP-β-CD) was dissolved in 100 mL of distilled water under continuous stirring to form solution A. Concurrently, 0.228 g of RES was dissolved in 2 mL of methanol and subjected to ultrasonic treatment for 30 min to obtain solution B. Solution B was then slowly added dropwise to solution A, followed by heating and stirring at 45°C and 800 r/min for 8 h. The temperature was subsequently reduced gradually to allow complete evaporation of methanol. The resultant solution (solution C) was filtered through a 0.45 μm filter membrane, and the filtrate was freeze-dried for 3 days to yield a white powder, identified as the RES inclusion complexes. To verify the formation of the inclusion complexes, a physical mixture of RES and HP-β-CD in equimolar ratio was prepared and used as a control.

#### Characterization of RES Inclusion Complexes

Four distinct sample groups were prepared for comprehensive characterization: the RES inclusion complexes, the physical mixture of RES and HP-β-CD, as well as individual samples of RES and HP-β-CD. FTIR spectra were recorded over the wavenumber range of 4000 to 400 cm^-1^ using an FTIR spectrometer. X-ray diffraction (XRD) patterns were obtained within the angular range of 5° to 70° using a Cu Kα diffractometer. Differential scanning calorimetry (DSC) was utilized to evaluate the thermal properties of each sample.

Furthermore, the thermal stability of the samples was evaluated by thermogravimetric analysis (TGA, Netzsch, Germany). Approximately 10 mg of each sample was placed in an alumina crucible and heated from 30°C to 500°C at a heating rate of 10°C/min under a nitrogen atmosphere (flow rate: 50 mL/min). The mass loss of the samples as a function of temperature was continuously recorded. In addition, the ¹H NMR analysis was conducted. The four sample groups were dissolved in deuterated dimethyl sulfoxide to prepare solutions, and measurements were carried out at room temperature using a nuclear magnetic resonance spectrometer (Bruker, Germany).

To evaluate the enhancement of RES water solubility by inclusion complexes in a simulated inflammatory environment, we conducted drug release experiments with the complexes. Equal volumes of free RES or RES inclusion complexes were placed in dialysis bags (1000 Da) and immersed in H₂O₂ solutions of varying concentrations. At predetermined time intervals, 200 μL of release medium was sampled and an equal volume of freshly prepared PBS containing the corresponding concentration of H₂O₂ were added. Absorbance was measured at 306 nm using a UV-visible spectrophotometer to quantitatively determine the cumulative release of RES.

### Fabrication and Characterization of Double-Layer Drug-Loaded MN@DOX+RES

#### Fabrication of MN@DOX+RES

In the initial phase, the tips layer and backing layer gels were prepared separately. Specifically, the lyophilized RES inclusion complexes powder was dissolved in PBS, and then used to prepare AHA-PBA/PVA hydrogel containing RES inclusion complexes as described above, which served as the formulation for the tips layer. HA and DOX were dissolved in PBS to prepare the backing layer gel.

In the subsequent phase, the microneedles were fabricated using a polydimethylsiloxane (PDMS) mold (array: 15×15; height: 800 μm) through a process involving vacuuming and centrifugation. First, the prepared drug-loaded AHA-PBA/PVA hydrogel was added into the PDMS mold, followed by vacuum treatment for 5 min using a vacuum pump to ensure the gel reached the needle tips. The mold was then centrifuged at 5000 rpm for 5 min to remove air bubbles. After drying for 6 h, the backing layer gel was added. Finally, the mold was placed in a drying oven for 72 h, and the microneedle patch (designated MN@DOX+RES) was carefully demolded. Each MN@DOX+RES patch contained approximately 100 mg of RES inclusion complexes and 1 mg of DOX.

Additionally, three types of MN were prepared using the same method for subsequent experimentation: drug-free MN (Blank MN), MN with drug loaded solely in the tips layer (MN@RES), and MN with drug loaded only in the backing layer (MN@DOX).

#### Morphological Characteristics of MN@DOX+RES

The macroscopic morphology of the MN@DOX+RES was examined using a stereo microscope (SOPTOP, China). The microstructure was characterized by SEM (ZEISS Sigma 360). To visualize the double-layer structure of the layered MN more distinctly, the tips layer was stained with Rhodamine B dye solution, while the backing layer was stained with Thioflavin T dye solution. After demolding, the patch was further visualized using a confocal laser scanning microscope (CLSM, Olympus, Japan).

#### Skin Insertion Capability of MN@DOX+RES

To evaluate the skin-piercing performance of MN@DOX+RES, initial insertion experiments were conducted on the dorsal skin of live Sprague-Dawley (SD) rats. Firstly, the dorsal hair was removed and the area disinfected. The microneedle patch was then applied for 30 s and subsequently removed. The skin surface was observed and documented at various time intervals until full recovery. To further verify the insertion capability, dorsal skin samples were excised, sectioned into smaller pieces, and the subcutaneous fat and excess fascia were carefully removed. The patch was re-applied to the excised skin and examined from the inner side. Additionally, hematoxylin and eosin (H&E) staining was performed to confirm successful insertion.

### Mechanical Strength Test

The mechanical strength of the MN@DOX +RES patch was evaluated by a universal testing machine (INSTRON, Model 5965, USA). Each sample was firmly affixed to a horizontal platform with the microneedle tips facing upward. A compression test was conducted by the load cell at a constant speed of 0.1 mm/s, and the resulting force-displacement curve was recorded in detail.

### *In Vitro* Degradation Detection

To evaluate the ROS-responsiveness of tips layer of the MN patches, uniformly sized patches were prepared using AHA-PBA/PVA hydrogels and immersed in 1 mL PBS solutions containing 0, 100, and 500 μM H₂O₂. The uniformly swollen mass was recorded as the initial degradation mass. Then, samples were retrieved on days 1, 2, and 3 for weighing, and residual mass (%) was calculated. Simultaneously, the morphology of the tips was observed and photographed, and the degradation characteristics of double-layer MN were recorded by shooting videos under a stereomicroscope.

### *In Vitro* Drug Release

MN@RES was individually incubated in PBS solutions containing 0 μM, 100 μM, and 500 μM H_2_O_2_ under continuous stirring at 200 rpm and maintained at 37°C. At predetermined time intervals, 200 μL of the release medium was sampled, and an equivalent volume of freshly prepared PBS solution containing the corresponding concentration of H₂O₂ was added. The cumulative release of RES was quantified by measuring the absorbance at 306 nm using a UV-vis spectrophotometer. Similarly, MN@DOX were incubated in PBS, and 200 μL of the medium was collected at scheduled intervals and replaced with an equal volume of fresh PBS. The release of DOX was monitored by measuring the absorbance at 350 nm.

### Biocompatibility Test

#### Cytocompatibility

The *in vitro* cytocompatibility of the MN@DOX+RES was evaluated using L929 fibroblasts and RAW264.7 macrophages through Calcein-AM/PI double staining and CCK-8 assays. MN samples were incubated in serum-containing culture medium to obtain the extracts. L929 and RAW264.7 cells were seeded into 24-well plates and co-cultured with the extracts for 24, 48, and 72 h. To assess cell viability, Calcein-AM/PI double staining was conducted. After incubation, the cells were washed three times with PBS and stained with 400 μL of the staining solution according to the manufacturer's instructions. After a 30-min incubation in the dark, the cells were examined under a fluorescence microscope (Leica, DMi8, Germany).

Subsequently, the CCK-8 assay was performed to quantitatively evaluate cell viability. L929 and RAW264.7 cells were seeded into 96-well plates (1 × 10^4^ cells per well) and cultured in DMEM supplemented with 10% FBS and 1% penicillin-streptomycin at 37°C in a humidified atmosphere containing 5% CO_2_. After 24 h of incubation, the medium was replaced with the microneedle extracts, and cell viability was measured on days 1, 2, and 3. Briefly, a working solution was prepared by diluting the CCK-8 reagent to 10% (v/v) in culture medium. Then, 100 μL of this solution was added to each well, followed by a 2-h incubation in the dark. Absorbance was measured at 450 nm using a microplate reader (TECAN Infinite M200 PRO, China).

#### Hemocompatibility

The prepared samples were immersed in 10 mL PBS to obtain the extracts. Fresh blood (1 mL) was collected from SD rats and washed three times with PBS. After discarding the supernatant, the red blood cells were resuspended in 10 mL of PBS. Then, 200 μL of the red blood cell suspension was transferred into centrifuge tubes, followed by the addition of 800 μL of PBS (negative control), double-distilled water (ddH_2_O, positive control), or the sample extracts, respectively. All groups were incubated at 37°C for 2 h. The absorbance at 545 nm was measured by a microplate reader spectrophotometer (TECAN Infinite M200PRO, China). The morphology of the red blood cells was captured by a fluorescence microscope. The hemolysis rate was calculated as follows:

Hemolysis Ratio (%) = [(A sample - A negative) / (A positive - A negative)] × 100%

Where A represents the absorbance.

### *In Vitro* Antimicrobial Activity

The antibacterial efficacy of MN@DOX +RES patches was validated using *Staphylococcus aureus (S. aureus)* and* Escherichia coli (E. coli)* as representative bacterial strains. The assessment included plate counting, live/dead staining of free bacteria, and bacterial biofilm analysis.

Bacterial suspensions of *S. aureus* or *E. coli* (1×10^6^ CFU/mL) were prepared and added to centrifuge tubes, which were subsequently randomly allocated into five groups. The control group was cultured in standard Luria-Bertani (LB) medium, whereas the experimental groups were co-cultured in LB medium supplemented with Blank MN, MN@DOX, MN@RES, or MN@DOX+RES samples, respectively. After 2 h, 100 μL of the bacterial suspension diluted with PBS was evenly spread onto LB agar plates and subsequently incubated at 37°C for 24 h. Colony-forming units (CFUs) were counted and imaged for each group. Additionally, the surface morphology of *S. aureus* and *E. coli* from the control and MN@DOX + RES groups was further characterized using SEM. Moreover, the bacterial suspensions from each group were collected in centrifuge tubes, followed by staining of *S. aureus* or *E. coli* with the live/dead bacteria dual-staining dye solution. A fluorescence microscope (Leica DMi8, Germany) was used to obtained the images.

To further assess the antibacterial efficacy of MN@DOX+RES on bacterial biofilms, *S. aureus* and *E. coli* were individually inoculated into 24-well plates at a concentration of 1×10^6^ CFU/mL. The plates were then incubated in a microbial culture incubator for 48 h to facilitate biofilm formation. Following this, each well was treated and grouped according to the aforementioned description. After 12 h of treatment, the medium was carefully removed, and the wells were washed twice with PBS. Subsequently, a dual-fluorescence live/dead bacterial staining solution was added to each well, and incubated in the dark for 15 min. The bacterial biofilms were then visualized, and images were captured and documented using a CLSM (Olympus, Japan).

### Antioxidant Capacity

#### Free Radical Scavenging Ability

The antioxidant properties of the MN@DOX+RES patches were evaluated through DPPH, ABTS, ·OH, H₂O₂, and ·O₂⁻ radical scavenging assays. Specifically, the DPPH working solution was prepared according to the manufacturer's instructions. The working solution was then mixed with the extract of the MN samples and incubated in the dark for 30 min. Absorbance was subsequently measured at 515 nm using a UV-vis spectrophotometer. For the ABTS assay, 100 μL of the extracts was added to 1 mL of ABTS working solution in a centrifuge tube, followed by incubation in the dark for 30 min. Absorbance was subsequently measured at 734 nm. The ·OH scavenging capacity was assessed using the salicylate method by measuring the absorbance of the reaction mixture at 510 nm. The H₂O₂ and ·O₂⁻ scavenging activities were determined by monitoring the absorbance decrease at 240 nm and the inhibition of pyrogallol autoxidation at 325 nm, respectively. The scavenging efficiency of them were calculated based on the provided instructions.

#### Intracellular Antioxidant Capacity

To evaluate the protective efficacy of the MN@DOX+RES patch against H_2_O_2_-induced oxidative stress, a DCFH-DA fluorescent probe and cytoskeletal staining were employed. L929 cells were seeded in 24-well plates at a density of 3×10^4^ cells per well and cultured in DMEM supplemented with 10% FBS and 1% penicillin-streptomycin for 12 h. Then, the culture medium was replaced with extracts containing different microneedle samples. After 24 h, 100 μM H_2_O_2_ was added, followed by incubation for 2 h. The cells were washed once with PBS, and then 400 μL of DCFH-DA working solution was added to each well. After incubation in the dark for 30 min, fluorescence images were acquired using a fluorescence microscope and analyzed with ImageJ software.

Moreover, to visualize cytoskeletal changes in L929 cells after the above treatments, the cells were fixed with 4% paraformaldehyde for 15 min and subsequently stained with FITC-labeled phalloidin and DAPI. Cytoskeletal morphology was then observed under a fluorescence microscope.

In addition, RAW264.7 cells were seeded into 12-well plates at a density of 4×10^5^ cells per well. The cells were treated following the same procedure described above, and then incubated with DCFH-DA working solution in the dark for 30 min. To evaluate the ROS-scavenging ability of the MN samples in RAW264.7 cells, fluorescence intensity was quantified using a multimodal small animal *in vivo* imaging system (ABL X6, Tanon, China).

### Immunomodulatory Effect of MN@DOX+RES on RAW264.7 cells

To evaluate the *in vitro* immunomodulatory activity of MN@DOX+RES patches, immunofluorescence staining and flow cytometry analysis were conducted. Macrophage polarization was initially evaluated through immunofluorescence staining by detecting the expression of CD206 (a mannose receptor) and inducible nitric oxide synthase (iNOS).

RAW264.7 cells were seeded into 24-well or 6-well plates. After 12 h, the experimental group was stimulated with lipopolysaccharide (LPS, 100 ng/mL; Sigma-Aldrich, USA). After 24 h of LPS stimulation, the cells were incubated with culture medium containing extracts from different MN samples for an additional 24 h, while the control group received standard culture medium without any treatment.

The cells were then washed with PBS and fixed with 4% paraformaldehyde for 30 min. Membrane permeabilization was performed using 0.5% Triton X-100 for 10 min, followed by blocking with 5% goat serum for 1.5 h at room temperature. Primary antibodies—anti-iNOS (1:100; Proteintech, China) or anti-CD206 (1:100; Proteintech, China)—were added to each well (250 μL), and incubated overnight at 4°C. The following day, the plate was equilibrated to 25°C for 30 min and incubated with a goat anti-rabbit IgG secondary antibody (1:200) for 1 h in the dark. Cell nuclei were stained with DAPI for 10 min, and the stained cells were visualized using a fluorescence microscope (Olympus, Japan). Fluorescence intensity was quantified using ImageJ software.

In addition, flow cytometry was performed to further quantify the proportion of macrophage phenotypes. RAW264.7 cells were treated as described above, harvested, washed, and incubated with anti-iNOS and anti-CD206 antibodies in the dark for 30 min. Flow cytometry (ACEA NovoCyte^TM^ 2070R, USA) was used to analyze the samples, and FlowJo software was used for data processing.

### *In Vitro* Cell Migration and Tube Formation Assays

The Transwell migration assay and scratch wound healing assay were performed to assess the effectiveness of MN@DOX+RES in promoting cell migration. In the Transwell assay, HUVECs were seeded in the upper chambers at a density of 2 × 10^4^ cells per well in 100 μL of serum-free medium. The lower chambers were filled with 650 μL of complete medium containing extracts from the respective MN samples. After 24 h of incubation, non-migrated cells on the upper surface of the membrane were carefully removed using a cotton swab. The migrated cells on the underside were then fixed in 4% paraformaldehyde for 30 min and rinsed with PBS. Following fixation, the cells were stained with 0.5% crystal violet solution for 30 min. Finally, the stained cells were observed under a light microscope, and migrated cells were quantified using ImageJ software.

For the scratch wound assay, HUVECs were seeded in 6-well plates at a density of 5 × 10^5^ cells per well. After reaching 90% confluence, a straight scratch was created using a sterile pipette tip. Detached cells were removed by PBS washing. Cells were cultured in DMEM supplemented with 2% FBS, and the experimental groups were treated with MN extracts. Scratch images were captured at 0, 12, and 24 h. The migration rate was calculated as:

[(A_0_ - A_t_)/A_0_] × 100%

Where A_0_ is the scratch area at 0 h and A_t_ is the remaining area at each time point.

Endothelial tube formation assays were conducted to evaluate the angiogenic capacity of MN@DOX+RES. Matrigel (100 μL) was added to pre-chilled 48-well plates and allowed to polymerize at 37°C for 1 h. HUVECs were then seeded at 2 × 10^4^ cells per well and incubated with 200 μL of MN extract solution. After 6 h, tube formation was observed under a light microscope, and ImageJ software was used to analyze the number of meshes, master junctions, and total tube length.

### *In Vivo* Drug Release of Double-layer MN

To visualize the release kinetics of double-layer MN *in vivo*, we conducted *in vivo* fluorescence imaging experiments. Cy5 was selected as the model drug, loaded onto the backing layer and tips layer to simulate DOX and RES release, respectively. These dye-loaded MN samples were applied to rat wound infection models. Imaging was performed at 0.5, 12, 24, 36, and 48 h post-application using an IVIS Lumina III imaging system (IVIS Spectrum, USA).

### Therapeutic Effect of MN@DOX+RES on Infected Wound Healing in Rats

All animal procedures were performed in accordance with the institutional guidelines and were approved by the Animal Ethics Committee of Southwest Medical University (Ethics Approval Number: 20241028-004). Infected wound models were established in adult male SD rats (200-220 g) to evaluate the therapeutic efficacy of MN@DOX+RES patches in promoting wound healing. Under anesthesia, a full-thickness circular skin wound (10 mm in diameter) was created on the dorsal surface of each rat. Subsequently, 100 µL of a *S. aureus* suspension (1 ×10^8^ CFU/mL) was injected to the wound to establish infection. 6 h post-inoculation, the rats were randomly allocated to five groups (n = 5 per group): (1) Control (no treatment); (2) Blank MN patch; (3) MN@DOX patch; (4) MN@RES patch; (5) MN@DOX+RES patch. Photographs of the wounds were taken on days 0, 3, 7, and 10. Wound exudates were collected on day 1, and skin tissues were harvested and fixed in 4% paraformaldehyde on days 3, 7, and 10 for histological and immunohistochemical analyses, including H&E staining, Masson's trichrome staining, immunofluorescence staining (markers: CD31, α-SMA, CD86, CD206, Nrf2, HO-1) and immunohistochemical staining (markers: IL-6, IL-10).

### Anti-inflammatory and antioxidant mechanisms of MN@DOX+RES

First, immunofluorescence staining was employed to assess the expression levels of nuclear factor erythroid-2 related factor 2 (Nrf2) and heme oxygenase-1 (HO-1). RAW264.7 macrophages were seeded in 6-well plates and incubated for 12 h. Cells were then stimulated with 100 ng/mL LPS (Sigma Aldrich, USA) for 24 h, followed by treatment with MN extracts for an additional 24 h. The control group was cultured under standard conditions without any treatment. After treatment, cells were incubated with primary antibodies against Nrf2 (1:100; Cell Signaling Technology, USA) and HO-1 (1:100; Cell Signaling Technology, USA) according to established immunofluorescence protocols. Fluorescence images were captured using a fluorescence microscope, and 3D heatmaps were generated using ImageJ software.

Then, Western blot analysis was performed to determine the protein expression levels of Keap1, Nrf2, HO-1, and β-actin. RAW264.7 cells were subjected to the same treatment regimen as in the immunofluorescence experiments. The cells were lysed using RIPA buffer (Epizyme Biotech, China) and kept on ice for 10 min. Lysates were then sonicated and centrifuged at 12,000 rpm for 10 min at 4 °C. The supernatant was collected and the protein concentration was quantified using a BCA Protein Assay Kit (Epizyme Biotech, China). Samples were mixed with 5× SDS-PAGE loading buffer (Beyotime, China) at a 4:1 ratio, boiled at 100 °C for 10 min, and stored at -80 °C. Proteins were separated by SDS-PAGE and transferred onto PVDF membranes. After blocking at room temperature for 1 h, membranes were incubated overnight at 4 °C with primary antibodies against Keap1 (1:5000), Nrf2 (1:2000), HO-1 (1:1000), and β-actin (1:5000) (all from Proteintech, China). The next day, membranes were incubated with a rabbit secondary antibody (1:5000; Proteintech, China) for 1 h at room temperature. Immunoreactive bands were visualized using an enhanced chemiluminescence (ECL) kit (Beyotime, China) and imaged with a BIO-OI gel documentation system (Optical Instrument Biotechnology, China).

To further investigate the regulatory role of Nrf2 pathway activation in modulating antioxidant and anti-inflammatory responses, quantitative real-time PCR (qRT-PCR) was conducted to quantify the mRNA expression levels of the antioxidant enzymes superoxide dismutase (SOD) and catalase (CAT), along with the pro-inflammatory cytokines interleukin-6 (IL-6) and interleukin-1β (IL-1β). RAW264.7 cells were grouped and treated as described in the immunofluorescence experiments. Total RNA was extracted using the SteadyPure Quick RNA Extraction Kit (Accurate Biotechnology, China) according to the manufacturer's instructions. Complementary DNA (cDNA) was synthesized using the Evo M-MLV RT Kit (Accurate Biotechnology, China). qRT-PCR amplification was performed using the CFX96 Touch Real-Time PCR Detection System (Bio-Rad, USA). β-actin served as the internal control gene, and the relative expression levels of target genes were calculated using the 2^-ΔΔCt^ method. Primer sequences used for amplification are listed in Table S1.

### Statistical Analysis

Data were obtained from a minimum of three independent experiments, and the results are presented as mean ± standard deviation (SD). A one-way analysis of variance (ANOVA) was employed to assess significant differences among multiple groups, followed by post-hoc tests where applicable. All statistical analyses were conducted using GraphPad Prism version 10.1. The significance levels are denoted as follows: ^*^P < 0.05, ^**^P < 0.01, ^***^P < 0.001, and ^****^P < 0.0001, while ns denotes *P* > 0.05, indicating no significant difference.

## Results and Discussion

### Characterization of AHA-PBA/PVA Hydrogels

In this study, we successfully developed AHA-PBA/PVA hydrogels capable of responding to ROS. As shown in Figure S1A, HA was first oxidized by sodium periodate to introduce aldehyde groups, which subsequently reacted with the amino group of 3-APBA through Schiff base formation to yield AHA-PBA. The chemical modification process is schematically illustrated. AHA-PBA was then crosslinked with PVA *via* dynamic boronate ester bonds, resulting in the formation of AHA-PBA/PVA hydrogels (Figure S1B). As illustrated in Figure S1C, the physical appearance of the hydrogel changed notably before and after crosslinking. Notably, this synthesis strategy eliminated the need for chemical crosslinking agents (e.g., EDC/NHS 32,33), offering a more environmentally friendly and milder alternative.

SEM imaging (Figure S1D) revealed that the AHA-PBA/PVA hydrogels exhibited a porous network structure, which facilitates exudate absorption and supports cell adhesion and metabolic exchange 34. UV-vis spectra (Figure S1E) showed that 3-APBA had absorption peaks at 208, 233, and 296 nm, while AHA-PBA exhibited red-shifted peaks at 217, 249, and 308 nm, indicating successful conjugation of 3-APBA onto the HA backbone. FTIR analysis (Figure S1F) confirmed the presence of aromatic C=C stretching vibrations (1485-1600 cm^-1^) and B-O characteristic peaks at 1338 cm^-1^ in AHA-PBA, further validating the incorporation of the phenylboronic acid structure 35. ^1^H NMR spectra (Figure S1G) of AHA-PBA displayed peaks between 6-8 ppm corresponding to aromatic protons 33,36. The degree of substitution of phenylboronic acid groups was calculated to be approximately 9.03%. Rheological analysis revealed that AHA-PBA/PVA hydrogels displayed typical viscoelastic behavior. As shown in Figure S1H, the storage modulus (G′) exceeded the loss modulus (G″) across a range of strains, indicating elastic-dominant properties. In the frequency sweep (Figure S1I), both G′ and G″ remained relatively constant, suggesting good structural stability of the hydrogel network. In addition, the shear viscosity (Figure S2) showed pronounced shear-thinning behavior, where viscosity decreased with increasing shear rate, indicating favorable injectability and dynamic flowability.

To investigate the degradation behavior of the AHA-PBA/PVA hydrogel, *in vitro* degradation experiments were performed in PBS containing 0, 100, and 500 μM H_2_O_2_. As shown in Figure S1J, the results showed that the degradation rate of the hydrogel increased under oxidative stress conditions, and was further accelerated with increasing concentrations of H_2_O_2_. Specifically, in the absence of H_2_O_2_, the hydrogel network was completely degraded by day 13. In contrast, in 500 μM H_2_O_2_, the degradation time was shortened to 8 days, representing an accelerated degradation rate of approximately 1.6-fold. This enhanced degradation is attributed to the cleavage of boronate ester (B-O-C) linkages by H_2_O_2_, resulting in the formation of boronic acid hydroxyl intermediates (B-OH) and disintegration of the hydrogel network. In inflammatory or oxidative stress-related microenvironments, ROS levels are markedly elevated. Among them, H_2_O_2_ serves as one of the major ROS, with concentrations typically ranging from 50 to 500 μM, and up to 1 mM in localized lesions 37. This targeted degradation behavior exhibits excellent compatibility with such pathological microenvironments, suggesting promising potential for AHA-PBA/PVA hydrogels in ROS-responsive intelligent drug delivery (e.g., on-demand release at inflamed sites) and tissue regeneration under oxidative stress conditions.

Integrating structural characterization, rheological analysis, and degradation performance, the successfully fabricated AHA-PBA/PVA hydrogel not only achieved controllable crosslinking but also exhibited remarkable ROS-responsive degradation capability. These properties position it as a promising candidate for efficient drug delivery in oxidative stress-prone microenvironments.

### Characterization of RES Inclusion Complexes

Given the inherent challenge of incorporating hydrophobic drugs into hydrophilic microneedle matrices, improving the aqueous solubility of poorly water-soluble compounds is critical for achieving their therapeutic efficacy. RES, a hydrophobic polyphenolic compound, exhibits poor water solubility, limiting its direct and uniform incorporation into hydrogel systems 38. To address this issue, HP-β-CD, which possesses a hydrophobic inner cavity and a hydrophilic outer surface, was employed to form a host-guest complex with RES. The aromatic ring of RES fits into the hydrophobic cavity of HP-β-CD, resulting in the formation of the RES inclusion complexes, which significantly enhances the solubility and physicochemical stability of RES 31,39. Therefore, in this study, we first prepared the RES inclusion complexes to facilitate its dispersion in aqueous media and ensure efficient encapsulation in the microneedle delivery system.

To confirm the successful formation of the RES inclusion complexes, FTIR, XRD, DSC, TGA, and ¹H NMR analyses were conducted on four samples: RES, HP-β-CD, their physical mixture, and the RES inclusion complexes. Firstly, FTIR spectroscopy was used to confirm the formation of the RES inclusion complexes (Figure S1K). The spectrum of RES (curve a) exhibited a broad absorption band at 3293 cm^-1^, corresponding to the O-H stretching vibration. Characteristic aromatic C=C stretching bands were observed at 1587, 1511, and 1447 cm^-1^, along with bands at 1384 and 1150 cm^-1^ assigned to phenolic O-H and C-O stretching, respectively. A peak at 964 cm^-1^ was attributed to =C-H out-of-plane bending, indicative of olefinic groups. In contrast, the spectrum of HP-β-CD (curve b) showed a broad O-H stretching band at 3399 cm^-1^, a C-H stretching band at 2929 cm^-1^, and peaks at 1157 and 1029 cm^-1^ associated with C-O and C-O-C vibrations, while 947 and 850 cm^-1^ corresponded to glucopyranose skeletal modes. The physical mixture (curve d) exhibited a simple overlay of RES and HP-β-CD spectra, indicating no interaction. In comparison, the inclusion complexes (curve c) showed the disappearance of RES peaks at 1447 and 964 cm^-1^, along with shifts in other characteristic bands, suggesting host-guest interactions 40. These spectral changes confirm that RES was successfully encapsulated within the hydrophobic cavity of HP-β-CD.

Secondly, XRD was performed to evaluate changes in crystallinity (Figure S1L). The XRD pattern of RES (curve a) exhibited multiple sharp diffraction peaks, reflecting its crystalline nature, while HP-β-CD (curve b) presented a broad amorphous halo centered around 18.7°. The physical mixture (curve d) retained the distinct crystalline peaks of RES, indicating the absence of interaction. In contrast, the inclusion complexes (curve c) displayed only a broad amorphous peak, with the crystalline reflections of RES completely disappearing. This transformation indicates that RES was molecularly dispersed within the HP-β-CD matrix, forming amorphous host-guest complexes, which aligns with previous reports on inclusion systems 40,41.

Then, DSC and TGA analyses were performed to further confirm the formation of RES inclusion complexes (Figure S1M and Figure S3). The DSC thermogram of RES (curve a) showed a sharp endothermic peak at 270 °C, corresponding to its melting point. HP-β-CD (curve b) exhibited a broad endothermic event around 90 °C due to water loss and the amorphous structure. The physical mixture (curve d) preserved the melting peak of RES, whereas the inclusion complexes (curve c) lacked any discernible endothermic peak associated with RES, indicating the loss of crystallinity and the successful formation of an amorphous inclusion complex. Consistently, the TGA curve revealed the disappearance of the RES decomposition peak (270-350°C) in the inclusion complexes (curve c), suggesting altered thermal stability and confirming the generation of a new phase distinct from the physical mixture (curve d).

Furthermore, the ¹H NMR spectra (Figure S4) showed a significant reduction in the characteristic proton signal intensity of RES in the inclusion complexes compared with the physical mixture. Meanwhile, the chemical shifts of protons (H-3 and H-6) within the HP-β-CD cavity exhibited notable changes, providing direct evidence for the encapsulation of RES molecules into the cyclodextrin cavity. Collectively, the results from FTIR, XRD, DSC, TGA, and ¹H NMR analyses corroborate the successful formation of the RES inclusion complexes.

Subsequently, we investigated the fundamental role of RES inclusion complexes in drug release. As shown in Figure S5, free RES exhibited slow and incomplete release in PBS, attributed to its inherent low water solubility. In stark contrast, the RES inclusion complexes showed a markedly accelerated and consistent release profile in all tested media (PBS with 0, 100, and 500 μM H_2_O_2_). These results demonstrate that the inclusion of RES into HP-β-CD significantly improves its aqueous solubility and maintains release stability under oxidative stress conditions.

### Morphological Characteristics of MN@DOX+RES

**Figure 1:**
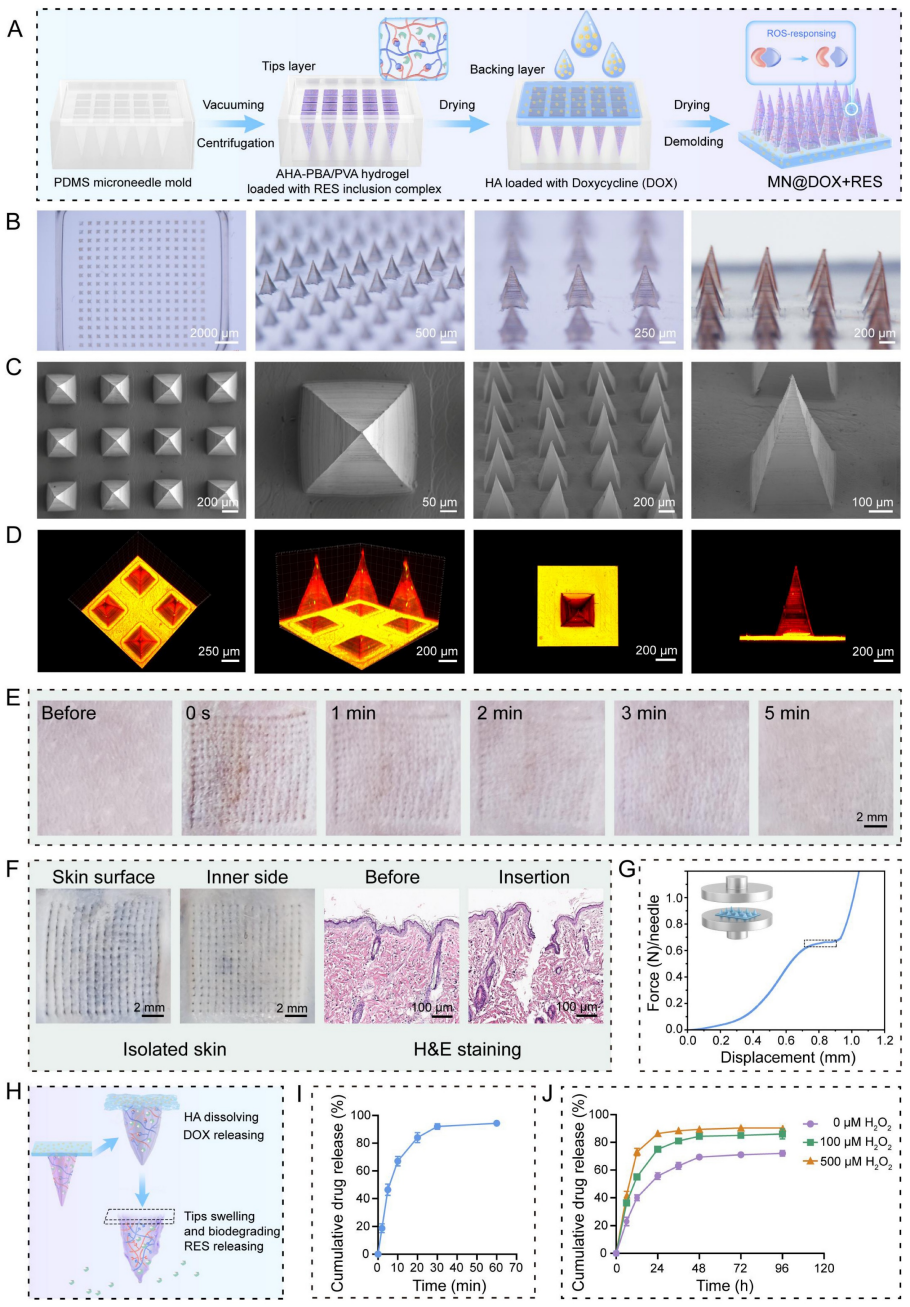
**Synthesis and Characterization of double-layer MN (MN@DOX+RES). (A)** Schematic illustration of the preparation of MN@DOX+RES patches. **(B)** Optical images of MN@DOX+RES observed under a stereo microscope (scale bar = 2000, 500, 250 and 200 μm). **(C)** SEM images of MN@DOX+RES (scale bar = 200, 100 and 50 μm). **(D)** Laser scanning confocal microscopy image showing the double-layer structure of MN@DOX+RES. **(E)** Skin recovery after MN@DOX+RES insertion observed macroscopically (scale bar = 2 mm). **(F)** Macroscopic and histological examination of skin penetration by MN@DOX+RES (scale bar = 2 mm and 100 μm). **(G)** Mechanical strength test of MN@DOX+RES. **(H)** Schematic diagram illustrating the layer-by-layer drug release mechanism of MN@DOX+RES. **(I)** *In vitro* cumulative drug release profile of DOX from MN@DOX in PBS (n = 3). **(J)** *In vitro* cumulative drug release profiles of RES from MN@RES in PBS containing 0, 100, and 500 μM H_2_O_2_ (n = 3).

MN@DOX+RES patches were fabricated by vacuum-assisted casting and centrifugation using PDMS molds (Figure 1A). Observation under a stereomicroscope revealed that each patch consisted of 225 microneedle tips arranged in a 15 × 15 array (Figure 1B). The MN tips exhibited a quadripyramidal morphology and were uniformly distributed on the backing layer in an orderly manner. Images obtained from the stereomicroscope, SEM (Figure 1C), and CLSM (Figure 1D) further confirmed that each MN had a sharp, straight, and pyramid-like structure, enabling effective skin penetration, resembling the function of a syringe needle. To visualize the dual-layer architecture, Rhodamine B and Thioflavin T were used to stain the tips layer and backing layer, respectively, enabling a clear and intuitive visualization of the double-layered structure (Figure 1D).

### Skin Tissue Puncture Performance and Mechanical Strength of MN@DOX+RES

Sufficient mechanical strength is a prerequisite for the successful application of MN. MN@DOX+RES patches were inserted into the dorsal skin of live SD rats and excised rat skin to evaluate their skin puncture capability. After inserting the patch array into the living rat skin for 30 s, corresponding micropores to the patch array were observed on the skin surface, which then disappeared within 5 min without any adverse reactions, such as erythema, edema, or bleeding, indicating excellent skin compliance of MN@DOX+RES (Figure 1E). Moreover, the MN tips were clearly visible from the inner side of the excised skin. The results of H&E staining showed discontinuity in the stratum corneum, and the pores formed reached the dermis, indicating that the patches prepared could puncture the rat skin successfully (Figure 1F).

The universal testing machine was used to evaluate the mechanical strength of MN@DOX+RES. According to existing research, the force required for the skin barrier to fail with successful insertion was approximately 0.098 N/needle 42. The stress-displacement curve showed that each needle could withstand a force of approximately 0.65 N (Figure 1G). Therefore, the MN@DOX+RES effectively punctured the skin without encountering any fractures, making it suitable for transdermal drug delivery.

### *In Vitro* Degradation and Drug Release

To evaluate the degradation and drug release characteristics of the tips and backing layers in the MN@DOX+RES, *in vitro* degradation and release experiments were performed. The layered release mechanism is illustrated in Figure 1H. Specifically, the DOX loaded in the HA-based backing layer is rapidly released upon HA dissolution, while the RES in the AHA-PBA/PVA-based hydrogel tips layer is gradually released *via* hydrogel swelling and oxidative degradation. Upon application to the wound area, the HA backing layer of the MN@DOX+RES patch absorbs wound exudate and dissolves, thereby maintaining a moist wound environment. As shown in the real-time recording (Video S1, Supporting Information), *in vitro* results demonstrated that the HA-based backing layer dissolved after absorbing PBS, while the hydrogel tips remained intact, indicating distinct degradation rates between the tips and backing layers. We further conducted degradation experiments to evaluate the ROS responsiveness of the tips layer. The results showed that the AHA-PBA/PVA tips layer degraded more rapidly with increasing H_2_O_2_ concentration (Figure S6).

As shown in Figure 1I, the release profile of DOX in PBS exhibited a rapid release behavior, with almost complete release occurring within 30 min, due to the rapid dissolution of HA in aqueous media. In contrast, the release profiles of RES in PBS containing 0, 100, and 500 μM H_2_O_2_ (Figure 1J) revealed that the release rate of RES increased progressively with the elevation of H_2_O_2_ concentration, this attributes to the ROS-responsive degradation behavior of the AHA-PBA/PVA hydrogel. H₂O₂ is a representative ROS that can cleave borate ester bonds, leading to the formation of boronic acid derivatives and the restoration of hydroxyl groups on polymer chains, thereby accelerating hydrogel degradation and facilitating drug release.

Collectively, these results confirm that MN@DOX+RES achieves a desirable sequential release pattern, with DOX released rapidly to exert immediate antibacterial effects, followed by sustained RES release for prolonged antioxidant and anti-inflammatory activity.

### Cytocompatibility and Hemocompatibility

**Figure 2:**
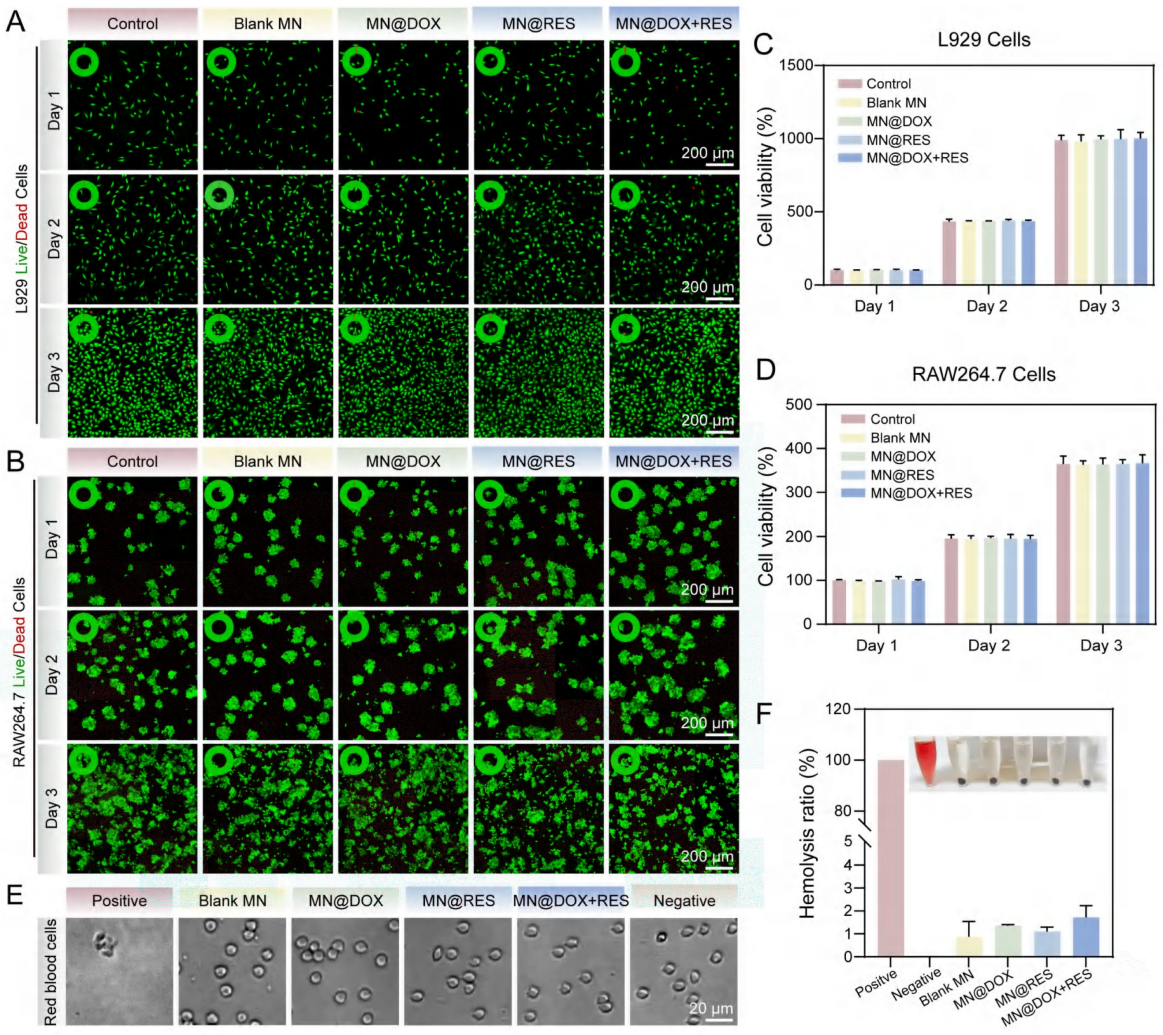
** Biocompatibility of the MN@DOX+RES. (A)** The Live/Dead staining images and **(B)** CCK-8 assays of L929 cells for 1, 2, and 3 days, respectively (scale bar = 200 μm, n = 3). **(C)** Quantitative analysis of CCK-8 assays of L929 cells for 1, 2, and 3 days, respectively (n = 3). **(D)** Quantitative analysis of CCK-8 assays of RAW 264.7 cells for 1, 2, and 3 days, respectively (n = 3). **(E)** Morphology of erythrocytes under the microscope. (scale bar = 20 μm, n = 3). **(F)** Picture of blood after co-culture with MN and Hemolysis rate of the MN (n = 3).

Excellent biocompatibility is an indispensable requirement for the successful application of biomaterials, as it significantly influences their *in vivo* stability, therapeutic efficacy, and long-term biosafety. To evaluate the biocompatibility of the MN@DOX+RES patch, Calcein-AM/PI double staining, CCK-8 assays, and hemolysis tests were performed *in vitro*. Live/Dead staining results (Figure 2A-B) showed predominantly green fluorescence (viable cells) and minimal red fluorescence (dead cells), confirming excellent cytocompatibility of the samples. This visual evidence of high cell viability aligns with the subsequent quantitative results. The results of the CCK-8 assay revealed no significant differences among the Control, Blank MN, MN@DOX, MN@RES, and MN@DOX+RES groups after 24 h, 48 h, and 72 h of cell culture (Figure 2C-D), indicated that MN prepared had no adverse effect on L929 and RAW264.7 cells.

Previous studies have showed that a hemolysis ratio lower than 5% 43 is regarded as satisfactory for hemocompatibility. As shown in Figures 2E-F, the positive control group exhibited a hemolysis rate of 100%, with only residual cell fragments visible microscopically. In contrast, the hemolysis rates for the Blank MN, MN@DOX, MN@RES, and MN@DOX+RES groups were all below 2%, well under the safety threshold, and intact red blood cells were observed, indicating negligible hemolytic activity. In a word, these findings demonstrate that MN@DOX+RES patches exhibit excellent biocompatibility and are suitable for biomedical applications.

### *In Vitro* Antimicrobial Activity

Bacterial infection substantially impedes wound healing through multiple mechanisms, including toxin secretion, immune suppression, metabolic disruption, and vascular damage 44,45. Among these, bacterial biofilm formation is particularly critical, as biofilms confer enhanced resistance to antibiotics and host immune responses, contributing to persistent infections and chronic wound development 46. Therefore, effective antibacterial strategies are essential to promote wound healing.

**Figure 3:**
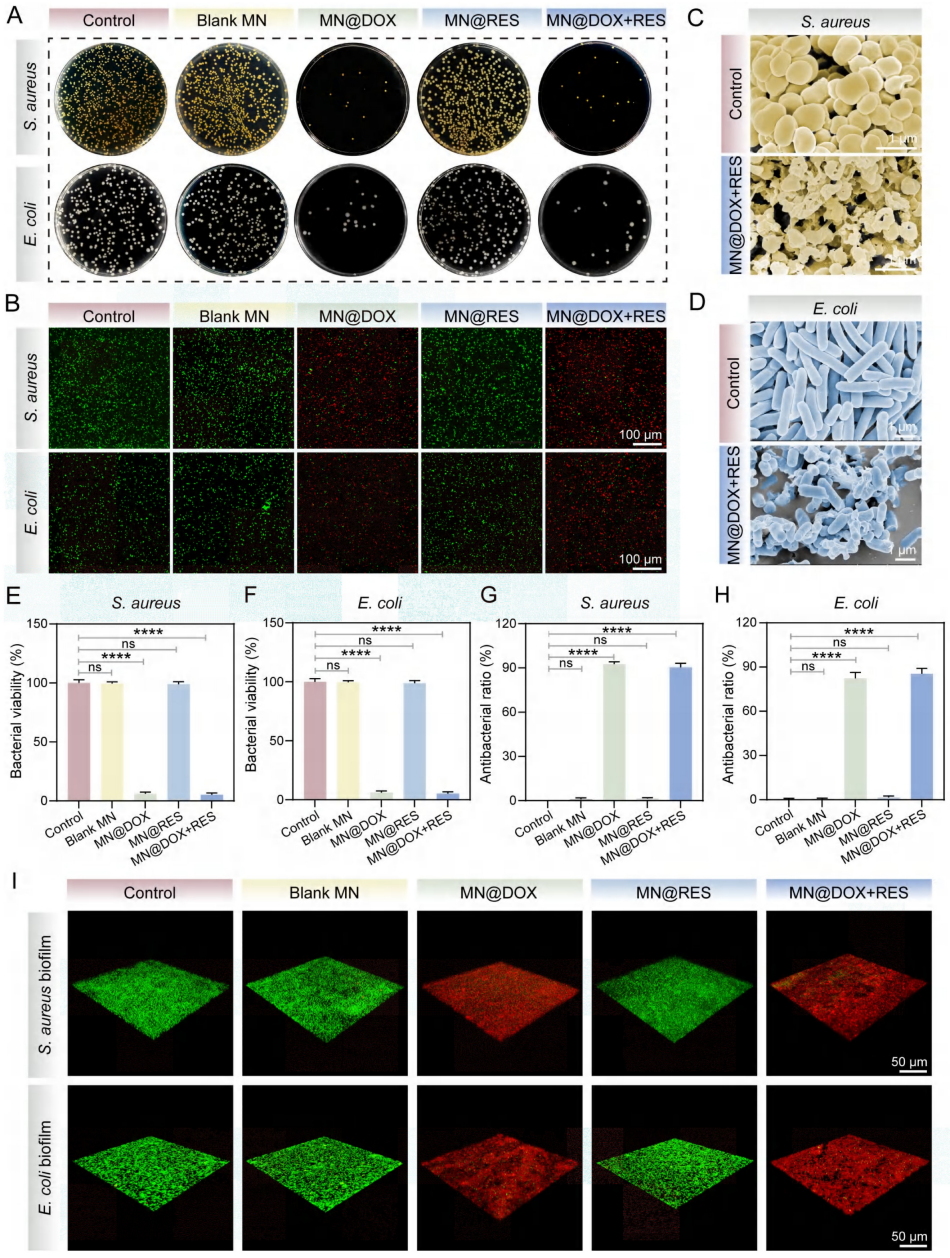
** Antibacterial bioactivities of MN@DOX+RES *in vitro*. (A)** Plate colony counting of *S. aureus* and *E. coli*, **(B)** Live/dead staining fluorescence images of bacterial biofilms of *S. aureus* and *E. coli*. Green, live bacteria; red, dead bacteria. (scale bar = 100 μm, n = 3). SEM images of the bacterial morphology of **(C)** *S. aureus* and **(D)** *E. coli.* (scale bar = 1μm, n = 3). Quantitative analysis of the bacterial viability of **(E)** *S. aureus* and **(F)** *E. coli*. Antibacterial ratio of **(G)** *S. aureus* and **(H)** *E. coli*. (n = 3) **(I)** Representative three-dimensional CLSM images and corresponding Z-stack projections of bacterial biofilms after Live/Dead staining (scale bar = 50 μm, n = 3). Statistical difference expression: ns, *p* > 0.05; ****p < 0.0001, n = 3.

The antibacterial performance of MN@DOX+RES was evaluated against *S. aureus* and *E. coli.* Plate counting results (Figure 3A, E-F) showed that the Blank MN and MN@RES groups had colony numbers comparable to the control, whereas the MN@DOX and MN@DOX+RES groups, both containing DOX, exhibited significantly reduced colony counts, indicating effective bacterial inhibition. To more intuitively investigate the effects of MN@DOX+RES on bacteria, the surface structural changes of the bacteria were examined using SEM. As shown in the Figure 3C-D, the bacteria in the control group retained intact, plump morphology, whereas the majority of bacteria treated with MN@DOX+RES displayed collapsed or even ruptured surfaces.

Furthermore, live/dead staining was used to assess bacterial viability. Viable bacteria fluoresced green, while dead bacteria appeared red, extensive red fluorescence was observed in the MN@DOX+RES group (Figure 3B). Quantitative analysis revealed that the antibacterial efficacy against *S. aureus* and *E. coli* exceeded 80%, demonstrating the strong antibacterial capability of the MN@DOX+RES (Figure 3G-H). In addition to planktonic bacteria, the antibiofilm effect of MN@DOX+RES was also evaluated. Three-dimensional CLSM imaging showed that biofilms in the MN@DOX and MN@DOX+RES groups were composed primarily of dead (red-stained) bacteria, while the control, Blank MN, and MN@RES groups showed predominantly viable (green-stained) bacteria (Figure 3I). These results are consistent with plate counting and live/dead staining of planktonic bacteria. In summary, these results confirm that DOX retains its antibacterial activity during MN fabrication, and the MN@DOX+RES patches exhibit potent antimicrobial properties.

### Free Radical Scavenging and Intracellular Antioxidant Properties

Excessive accumulation of ROS exacerbates cellular and tissue damage, significantly impeding the wound healing process 47. Therefore, bioactive materials with ROS-scavenging capabilities hold great promise in promoting tissue regeneration.

**Figure 4:**
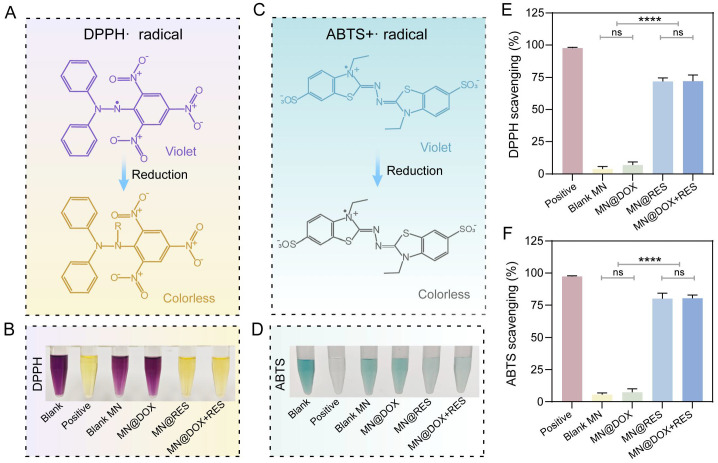
** Free radical scavenging ability of MN@DOX+RES *in vitro*.** Schematic representation of the scavenging process for **(A)** DPPH· and **(C)** ABTS+· free radicals. Photograph of the DPPH· radical **(B)** and **(D)** ABTS+· free radicals after incubation with MN extracts. Quantification of the DPPH· radical **(E)** and **(F)** ABTS+· free radicals scavenging rate. Statistical difference expression: ns, *p* > 0.05; ****p < 0.0001, n = 3.

The radical scavenging ability of the MN was evaluated using DPPH and ABTS assays. The DPPH radical, characterized by an unpaired electron, exhibits a deep purple color in solution. Upon donation of an electron from an antioxidant, the unpaired electron becomes stabilized, resulting in a color change from purple to yellow (Figure 4A). Spectrophotometric analysis indicated that the MN@DOX+RES group achieved a scavenging efficiency exceeding 70% (Figure 4B, E). Similarly, ABTS is oxidized to form the blue-green ABTS+ radical, which has a characteristic absorption at 734 nm. Antioxidants can reduce ABTS+ radicals, leading to a fading of the solution color (Figure 4C). The MN@DOX+RES group achieved an ABTS scavenging rate of approximately 80%, suggesting strong nitrogen-centered radical scavenging activity (Figure 4D, F). Photographs of different samples provided visual confirmation of this conclusion.

Additionally, we conducted scavenging experiments targeting hydroxyl radicals (·OH), hydrogen peroxide (H₂O₂), and superoxide anions (·O₂⁻). As shown in Figure S7, both the MN@RES and MN@DOX+RES groups exhibited significant scavenging activity. Notably, the highest scavenging efficiencies were observed for ·OH and ·O₂⁻, consistent with the highly reactive nature of ·OH and the ability of RES as an electron donor to effectively neutralize various free radicals. In contrast, the MN@DOX and Blank MN groups showed no significant scavenging capacity, further confirming that the antioxidant efficacy primarily originates from RES.

**Figure 5:**
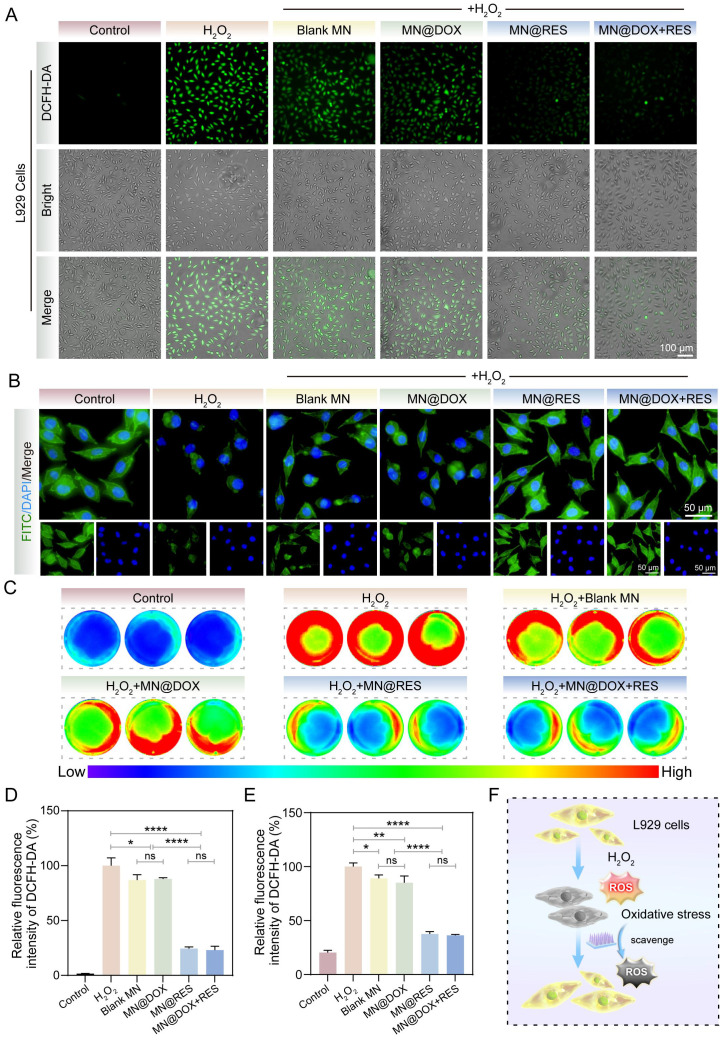
** MN@DOX+RES patches eliminate ROS in an oxidative stress environment. (A)** DCFH-DA staining images of L929 cells under H_2_O_2_-induced oxidative stress (scale bar = 100 μm, n = 3). **(B)** Cytoskeletal morphology of L929 cells under oxidative conditions visualized by FITC-labeled phalloidin staining (scale bar = 50 μm, n = 3). **(C)** Fluorescence images of ROS levels in H_2_O_2_-stimulated RAW264.7 cells after DCFH-DA staining. **(D, E)** Quantitative analysis of fluorescence intensity in L929 and RAW264.7 cells, respectively (n = 3). **(F)** Schematic diagram illustrating the antioxidative effects of MN@DOX+RES microneedles on L929 cells under oxidative stress. Statistical difference expression: ns, *p* > 0.05; *p < 0.05; **p < 0.01; ****p < 0.0001, n = 3.

To further validate the antioxidant efficacy at the cellular level, DCFH-DA fluorescence staining and cytoskeletal imaging were conducted. H_2_O_2_ treatment significantly increased intracellular ROS levels in L929 cells, as evidenced by strong green fluorescence in the positive control group (Figure 5A, D). A slight reduction in ROS levels was also observed in the Blank MN group, though not comparable to MN@RES or MN@DOX+RES. This may be attributed to the intrinsic properties of the hydrogel matrix 48. In contrast, the MN@RES and MN@DOX+RES groups exhibited markedly diminished fluorescence intensity, indicating effective ROS scavenging. Phalloidin staining further confirmed that MN@DOX+RES effectively alleviated H_2_O_2_-induced cytoskeletal damage, as evidenced by the preservation of spindle-shaped F-actin filaments and intact pseudopodia (Figure 5B). Similar trends were observed in RAW264.7 cells, where H_2_O_2_ stimulation led to elevated ROS levels (red), whereas treatment with MN@RES or MN@DOX+RES significantly reduced intracellular ROS levels, as indicated by diminished fluorescence intensity (blue/green) (Figure 5C, E). Previous studies have shown that the polyphenolic structure of RES can directly neutralize reactive oxygen species by donating hydrogen atoms or electrons 49. In addition, RES can indirectly enhance cellular resistance to oxidative stress by activating endogenous antioxidant pathways, such as the Nrf2/HO-1 signaling axis.

In conclusion, MN@DOX+RES demonstrated excellent antioxidant properties. It exhibited remarkable free radical scavenging capability *in vitro* and effectively mitigated cell damage induced by oxidative stress at the cellular level. These characteristics make it a promising candidate for alleviating oxidative stress-induced damage and facilitating wound healing.

### Immunomodulatory Effect

Chronic inflammation poses a significant challenge to wound healing. Macrophages, which function as critical regulators in the immune response, demonstrate a dual functionality during the wound healing process. Specifically, M1-type macrophages are primarily responsible for initiating inflammation and eliminating pathogens, whereas M2-type macrophages facilitate wound healing by resolving inflammation and promoting tissue regeneration 50,51. Therefore, we systematically evaluated the ability of MN@DOX+RES to modulate macrophage polarization by analyzing the expression of M1 and M2 phenotypic markers (iNOS and CD206) using immunofluorescence staining and flow cytometry *in vitro*.

**Figure 6:**
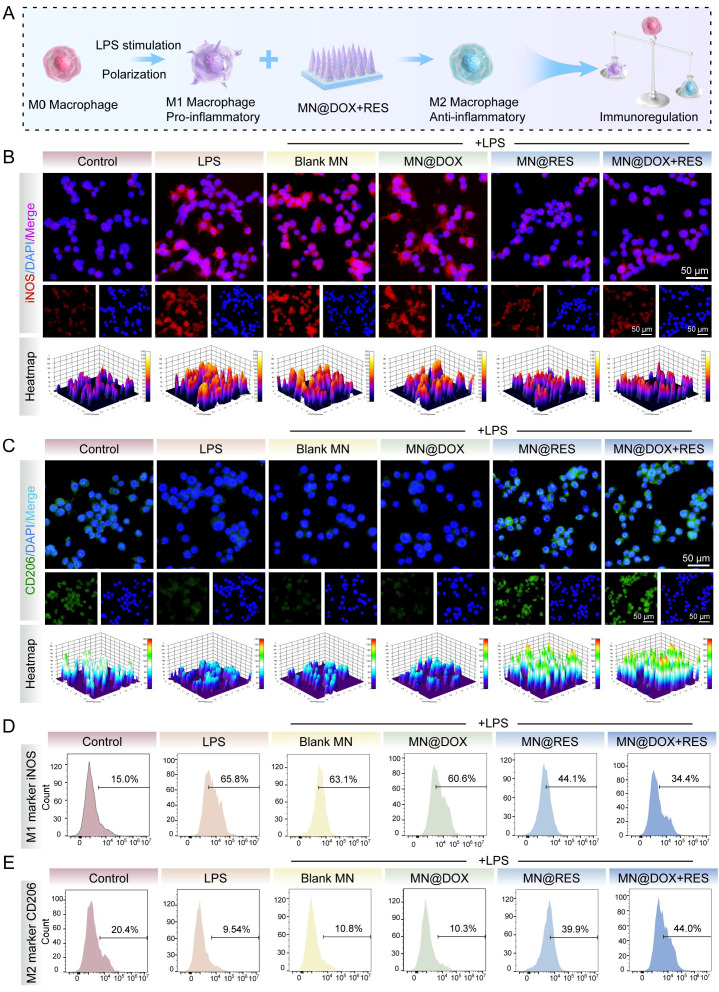
** Immunoregulatory properties of MN@DOX+RES patches on macrophages (A)** Schematic diagram illustrating that the MN@DOX+RES inhibit the polarization of macrophages toward the M1 phenotype and promote their polarization toward the M2 phenotype. Immunofluorescence images of **(B)** M1-type (iNOS) and **(C)** M2-type (CD206) macrophage biomarkers (scale bar = 50 μm, n = 3). Flow cytometry histograms of macrophage polarization markers: **(D)** iNOS for M1 macrophages and **(E)** CD206 for M2 macrophages, representative of three independent experiments (n = 3).

LPS stimulation drives macrophages toward an M1 phenotype, characterized by the release of pro-inflammatory mediators and elevated iNOS expression, thereby establishing an inflammatory microenvironment. In the immunofluorescence staining assay, compared with the LPS group, cells treated with MN@RES or MN@DOX+RES extracts exhibited a marked reduction in iNOS fluorescence intensity (Figure 6B) and a substantial increase in CD206 expression (Figure 6C). These results suggest that MN@RES and MN@DOX+RES may inhibit M1-type polarization while promoting the transition to the anti-inflammatory M2 phenotype.

To further quantify these effects, flow cytometry analysis was performed. In the LPS, LPS + Blank MN, and LPS + MN@DOX groups, the proportion of iNOS-positive macrophages was markedly elevated, reaching 65.8%, 63.1%, and 60.6%, correspondingly, compared to 15.0% in the untreated control group. However, the LPS + MN@DOX+RES group showed a substantial reduction to 34.4%, indicating suppressed M1 activation (Figure 6D). Conversely, the CD206-positive macrophage population remained low in the LPS, LPS + Blank MN, and LPS + MN@DOX groups (ranging from 9.54% to 10.8%), whereas it increased dramatically in the MN@RES and MN@DOX+RES groups, reaching 39.9% and 44.0%, respectively (Figure 6E). These findings demonstrate that MN@DOX+RES effectively promotes M2 macrophage polarization and attenuates the pro-inflammatory phenotype.

In summary, MN@DOX+RES exhibits robust immunomodulatory potential by regulating macrophage polarization. Through the downregulation of iNOS and upregulation of CD206, it promotes a phenotypic shift from M1 to M2 macrophages, thereby potentially alleviating chronic inflammation and creating a more favorable wound healing microenvironment.

### *In Vitro* Cell Migration and Tube formation Assays

**Figure 7:**
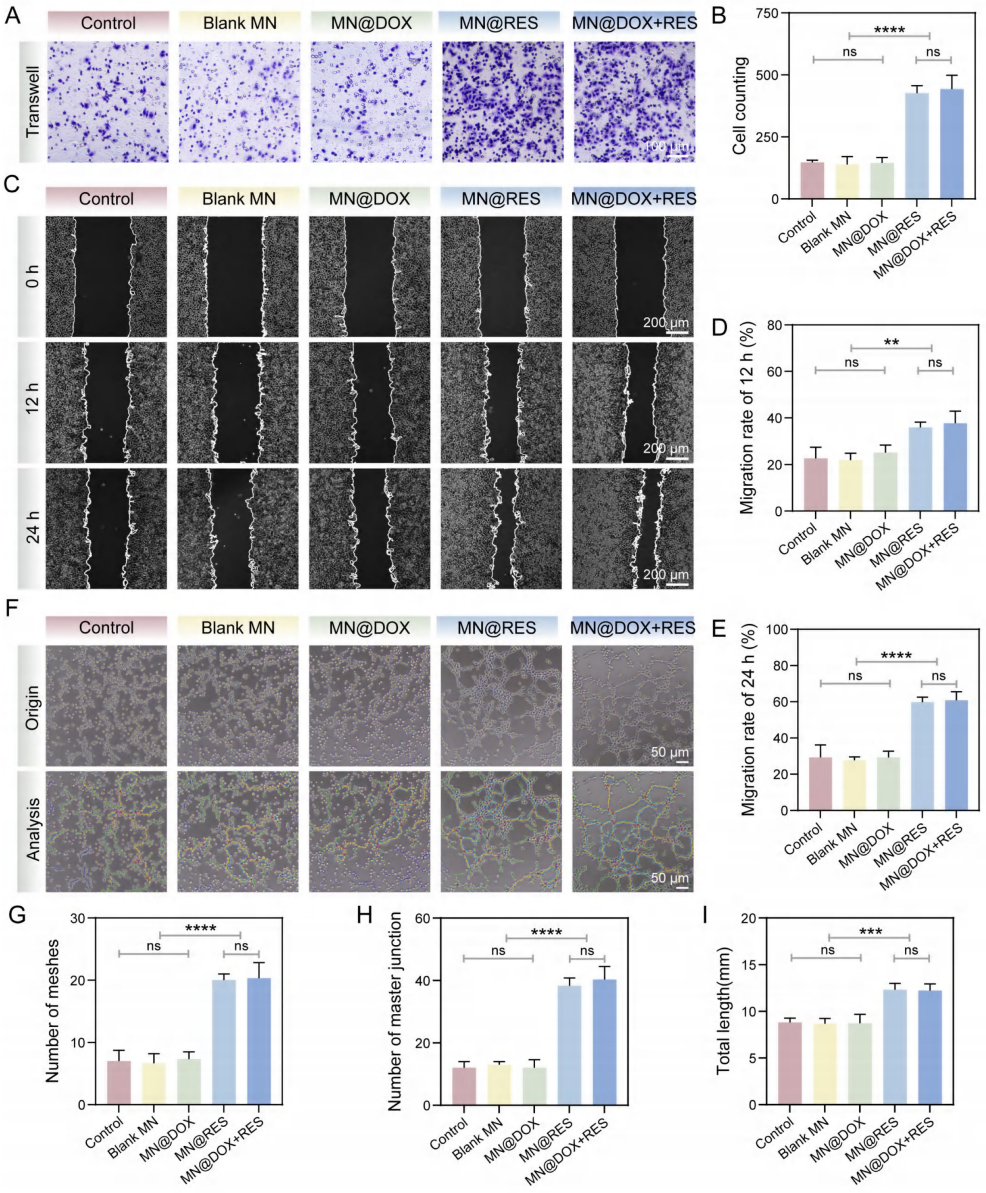
** MN@DOX+RES patches promote HUVECs migration and angiogenesis. (A-B)** Results of the effect of MN@DOX+RES on HUVECs migration by Transwell assay (scale bar = 100 μm, n = 3). **(C-E)** Results of the effect of MN@DOX+RES on HUVECs migration by wound healing assay (scale bar = 200 μm, n = 3). **(F-I)** Results of the tube formation assay evaluating the proangiogenic capacity of MN@DOX+RES (scale bar = 50 μm, n = 3). Statistical difference expression: ns, *p* > 0.05; **p < 0.01; ***p < 0.001; ****p < 0.0001, n = 3.

**Figure 8:**
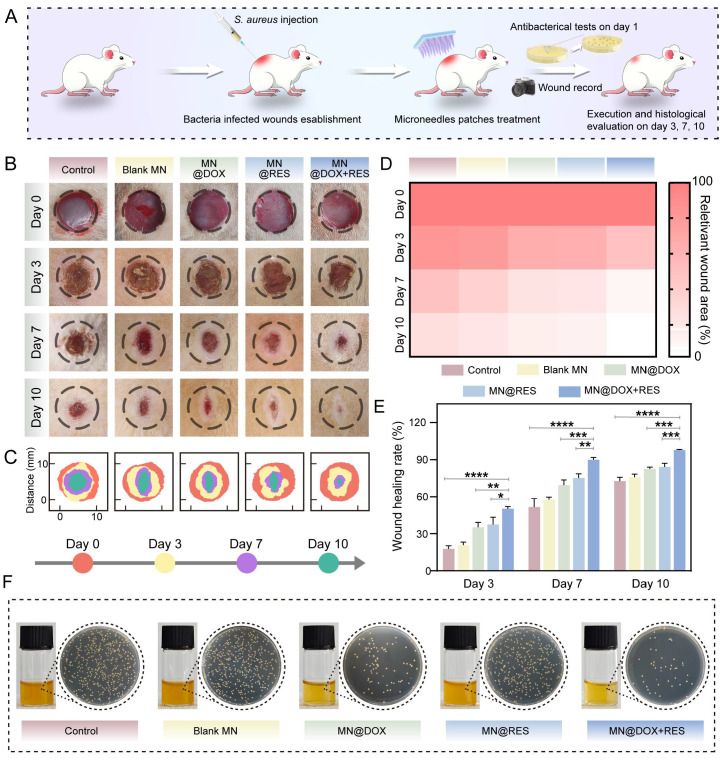
** MN@DOX+RES patches promote wound healing and antibacterial effects *in vivo*. (A)** Schematic diagram of the establishment of full-thickness infected wounds on the back of rats and the treatment procedure with MN@DOX+RES patches. **(B)** Representative wound healing images on days 0, 3, 7, and 10. **(C)** Schematic diagram illustrating the progress of wound healing areas over the course of 10 days under different treatment conditions. **(D)** Comparison of wound healing areas on days 0, 3, 7, and 10 for different MN treatments. **(E)** Quantitative data of the relative wound healing area at different time points (n = 5). **(F)** Representative colony images from wound exudates on day 1 after treatment with different MN samples (n = 5).

Wound healing is a complex biological process involving collective cell migration, which includes key events such as fibrin clot resolution, extracellular matrix (ECM) remodeling, collagen deposition, and wound contraction 52,53. In this study, the Transwell migration assay was first employed to assess the effects of MN@DOX+RES on cell motility. As shown in Figure 7A, cell migration in the Control, Blank MN, and MN@DOX groups was relatively limited. In contrast, the MN@RES and MN@DOX+RES groups exhibited significantly enhanced cell migration. Quantitative analysis revealed that the number of migrated cells in the MN@DOX+RES group was approximately threefold higher than that in the Control group (Figure 7B). Additionally, the scratch wound healing assay further validated the pro-migratory effects of MN@DOX+RES on HUVECs. As illustrated in Figure 8C, the initial wound area at 0 h was set as the reference, and the MN@DOX+RES group exhibited the highest wound closure rates at 12 and 24 h (Figure 7D-E). These results suggest that RES is the primary contributor to the observed enhancement in cell migration. RES has been widely reported to promote cell migration, which was further confirmed in this study.

Angiogenesis plays a pivotal role in wound healing by enhancing oxygen and nutrient supply, supporting blood flow for new tissue development, and accelerating wound healing 54. In the tube formation assay (Figure 7F), only limited tube formation was observed in the Control, Blank MN, and MN@DOX groups. In contrast, MN@RES and MN@DOX+RES treatments significantly increased the number of meshes, master junctions, and total tube length (Figure 7G-I). Overall, MN@DOX+RES markedly enhanced the *in vitro* migration and angiogenic potential of HUVECs, highlighting its promise for promoting angiogenesis and wound healing *in vivo*.

### *In Vivo* Drug Release of Double-layer MN

To further investigate the *in vivo* release behavior of the drug, we employed an IVIS Lumina III imaging system (IVIS Spectrum, USA) to monitor dye-loaded microneedles (Figure S9). Results showed that both the backing layer and the tips layer exhibited strong fluorescence signals in the early stages, diffusing toward the wound periphery within 12 h. However, their release patterns diverged significantly in the later stages: the dye in the backing layer faded almost 36 h after application. This indicates the backing layer enables rapid drug release, delivering high doses for early antimicrobial action. Conversely, the tips layer maintained detectable fluorescence intensity after 48 h, demonstrating sustained and prolonged drug release profile. These findings align with our *in vitro* release studies and support the synergistic therapeutic model of “rapid antimicrobial action coupled with sustained immunomodulation” observed in our experiments.

### Therapeutic Effect of MN@DOX+RES on Infected Wound in Rats

*S. aureus* is recognized as one of the predominant pathogenic bacteria associated with chronic wounds 55. Previous studies have indicated that full-thickness excisional skin wounds in rodent models (such as rats) are clinically relevant 56. Therefore, to assess the efficacy of MN@DOX+RES patches in promoting wound healing *in vivo*, we created a 10-mm-diameter full-thickness skin defect on the dorsal surface of each rat and established an infected wound model by inoculating the wound with *S. aureus*.

Photographic documentation and quantitative analysis of wound areas across the five groups on days 0, 3, 7, and 10 post-surgery are shown in Figure 8B-C. Notably, the MN@DOX+RES treatment resulted in a markedly enhanced wound healing rate compared to the other groups, as illustrated in Figure 8D-E. By day 7, the wound closure rate reached 50.3% in the control group, while the MN@DOX and MN@RES groups exhibited improved healing rates of 69.4% and 75.2%, respectively. Notably, the MN@DOX+RES group achieved a healing rate of 89.9%, highlighting its superior efficacy. By day 10, the skin defect in the MN@DOX+RES group had essentially closed, with only 2.1% of the wound remaining. To evaluate the *in vivo* antibacterial efficacy, wound exudates collected on day 1 were serially diluted with sterile PBS and subsequently plated onto LB agar for bacterial culture. Compared with other groups, both MN@DOX and MN@DOX+RES significantly reduced S. aureus colony formation (Figure 8F), indicating strong *in vivo* antibacterial activity.

**Figure 9:**
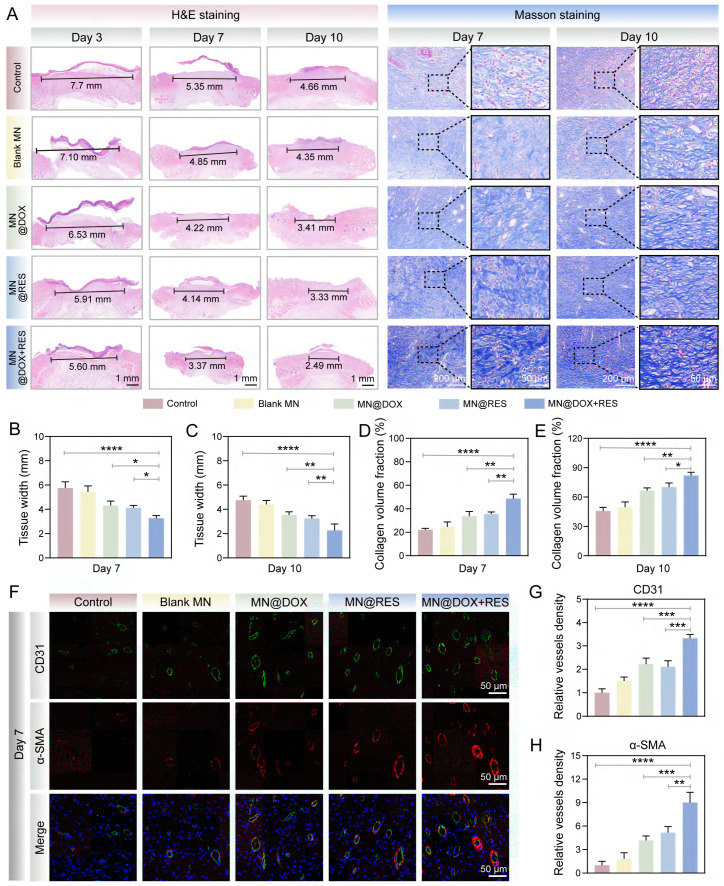
** Wound healing and angiogenesis verification after different MN treatments. (A)** Representative images of H&E staining and Masson staining on days 3, 7, and 10 (scale bar = 1 mm, 200 μm and 50 μm). **(B, C)** Statistical analysis of wound width on days 7 (B) and 10 (C). **(D, E)** Statistical analysis of collagen volume fraction on days 7 (D) and 10 (E). **(F)** Immunofluorescence staining of α-SMA and CD31 in wound tissue on the 7th day (scale bar = 50 μm). **(G, H)** Statistical analysis of relative vascular density on the 7th day. Statistical difference expression: *p < 0.05; **p < 0.01; ***p < 0.001; ****p < 0.0001, n = 5.

Wound tissues were collected on days 3, 7 and 10 for histological analysis. H&E staining was used to assess granulation tissue formation and reepithelialization, which are critical indicators of the reconstruction phase of wound healing. Granulation tissue formed between the wound edges, with a smaller gap indicating faster closure (Figure 9A). Epithelial cells migrated from the wound margins to promote reepithelialization. The MN@DOX+RES group showed the smallest granulation gap and was the first to complete reepithelialization (Figures 9B-C).

Masson's trichrome staining was employed to visualize collagen deposition, a critical factor in tissue remodeling. Collagen content increased over time, with day 10 showing loosely arranged collagen in the control group, while the MN@DOX, MN@RES, and MN@DOX+RES groups exhibited denser deposition. The MN@DOX+RES group showed the most compact and well-organized collagen bundles (Figure 9A). Quantification confirmed the highest collagen volume fraction in the MN@DOX+RES group on day 10 (Figures 9D-E), indicating superior tissue regeneration.

Given the essential role of angiogenesis in delivering oxygen and nutrients to regenerating tissues, we performed dual immunofluorescence staining for CD31 and α-SMA on day 7 to evaluate both neovascularization and vascular maturity. CD31 specifically labels vascular endothelial cells, reflecting vessel density, while α-SMA indicates the presence of smooth muscle cells or pericytes, markers of vascular maturation. On day 7, the control group showed sparse vascular structures with weak CD31 and α-SMA signals, whereas the MN@DOX and MN@RES groups demonstrated an increased number of vessels (Figure 9F). Notably, the MN@DOX+RES group exhibited the most extensive and mature neovascular networks, with the highest expression of both CD31 and α-SMA (Figures 9G-H), indicating robust angiogenesis.

**Figure 10:**
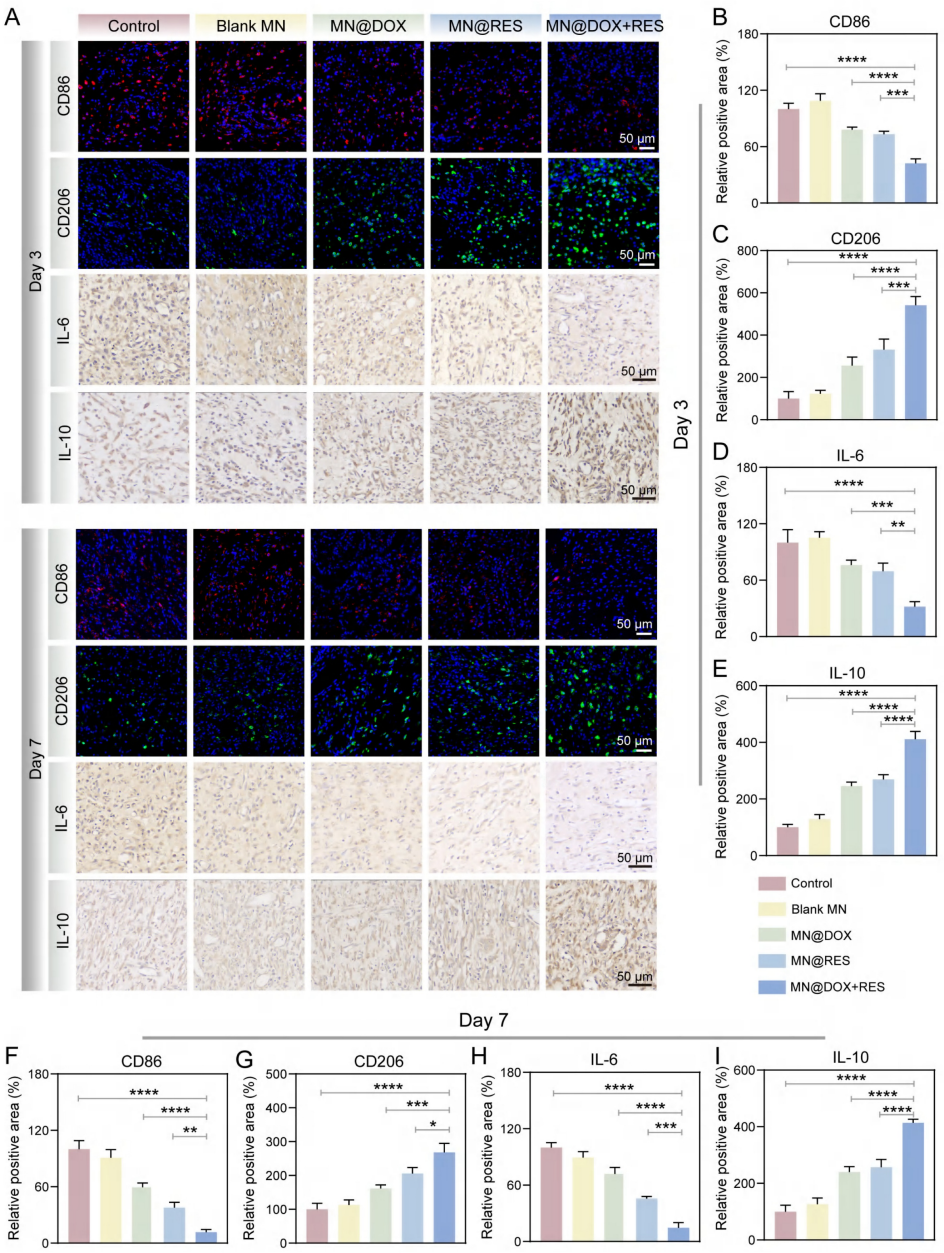
** Anti-inflammatory performance testing of MN@DOX+RES* in vivo*. (A)** Representative immunofluorescence images of CD86, CD206, IL-6 and IL-10 on days 3 and 7 after different treatments (scale bar = 50 μm). **(B-E)** Semi-quantitative analysis of the relative positive areas of CD86 (B), CD206 (C), IL-6 (D), and IL-10 (E) on day 3 after different treatments. **(F-I)** Semi-quantitative analysis of the relative positive areas of CD86 (F), CD206 (G), IL-6 (H), and IL-10 (I) on day 7 after different treatments. Statistical difference expression: *p < 0.05; **p < 0.01; ***p < 0.001; ****p < 0.0001, n = 5.

To comprehensively track the dynamic changes in macrophage polarization and cytokine profiles during the inflammatory and healing phases, immunofluorescence staining for CD86 (M1 marker) and CD206 (M2 marker) was performed on both day 3 and day 7, along with immunohistochemical staining for IL-6 and IL-10. As shown in Figure 10A, CD86-positive pro-inflammatory M1 macrophages were highly expressed in the control group, whereas MN@DOX+RES treatment markedly decreased CD86 expression on both days 3 and 7. In contrast, CD206 expression, indicative of anti-inflammatory M2 macrophages, was significantly upregulated in the MN@DOX+RES group at both time points (Figure 10B-C, 10F-G). These results suggest that MN@DOX+RES effectively promotes the phenotypic shift from M1 to M2 macrophage phenotypes, facilitating the resolution of inflammation and tissue regeneration.

In addition to cell phenotype markers, cytokine expression was examined to further validate the immunomodulatory effects. IL-6, a pro-inflammatory cytokine, was highly expressed in the control group but significantly suppressed in the MN@DOX+RES group on both days 3 and 7 (Figure 10A, 10D, 10H). Conversely, IL-10, an anti-inflammatory cytokine, showed substantially higher expression in the MN@DOX+RES group compared to controls (Figure 10A, 10E, 10I). These consistent findings across two key time points strongly indicate that MN@DOX+RES can bidirectionally modulate the inflammatory environment by downregulating pro-inflammatory cytokines while upregulating anti-inflammatory mediators.

### Anti-inflammatory and Antioxidant Mechanisms of MN@DOX+RES

When the body is infected, ROS levels increase dramatically. The excessive accumulation of ROS not only induces oxidative damage, thereby disrupting cellular homeostasis, but also triggers the release of pro-inflammatory cytokines such as IL-1β and IL-6, creating a vicious cycle between oxidative stress and inflammation 57. This cycle ultimately impedes the wound healing process. The Nrf2 signaling pathway plays an essential role in maintaining cellular redox balance and significantly contributes to alleviating oxidative stress and inflammatory responses 58,59. Under normal physiological conditions, Keap1 facilitates the proteasomal degradation of Nrf2 *via* a Cul3-dependent E3 ubiquitin ligase, thereby maintaining its low expression level. When cells are subjected to oxidative stress or other stimuli, the cysteine residues of Keap1 are oxidized, leading to its structural dissociation and the consequent release and activation of Nrf2. Subsequently, Nrf2 translocates to the nucleus, thereby promoting the transcription of antioxidant enzymes (e.g., HO-1) and genes linked to antioxidant response elements (ARE) 60,61. This mechanism enhances antioxidant capacity, suppresses inflammation, and reduces oxidative damage.

Previous studies have confirmed that RES can activate the Nrf2 pathway through various mechanisms, including disruption of the Keap1-Nrf2 interaction, upregulation of Nrf2 protein levels, promotion of antioxidant enzyme expression, and modulation of other related signaling cascades. These mechanisms collectively contribute to the antioxidant, anti-inflammatory, and cytoprotective properties of RES 62-64. Our experimental results demonstrate that both MN@RES and MN@DOX+RES significantly reduce intracellular ROS levels, inhibit macrophage polarization toward the pro-inflammatory M1 phenotype and promote polarization toward the anti-inflammatory M2 phenotype in an LPS-induced inflammatory environment.

**Figure 11:**
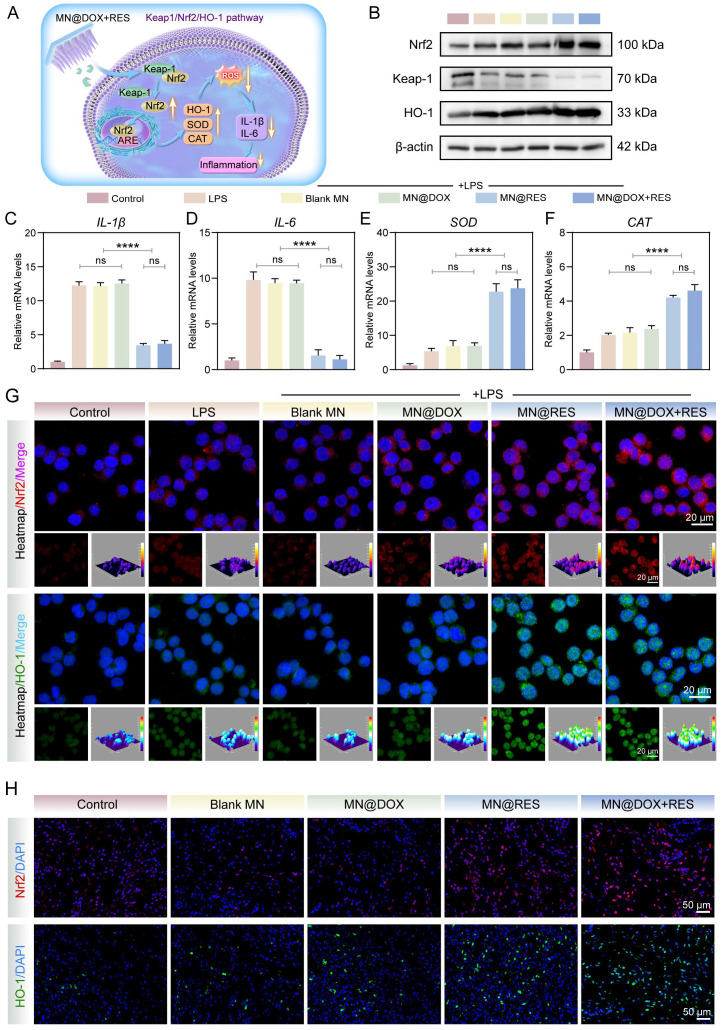
** Effects of MN@DOX+RES on the Keap1/Nrf2/HO-1 pathway. (A)** Schematic diagram of MN@DOX+RES clearing ROS and reducing inflammation through the Keap1/Nrf2/HO-1 pathway. **(B)** Expression of proteins related to the Keap1/Nrf2/HO-1 pathway. (n = 3). **(C-F)** Keap1/Nrf2/HO-1 pathway-related gene expression. Statistical difference expression: ns, *p* > 0.05; ****p < 0.0001, n = 3.** (G)** Immunofluorescence was used to detect the expression of Nrf2 and HO-1 in RAW264.7 (scale bar = 20 μm, n = 3). **(H)** Immunofluorescence was used to detect the expression of Nrf2 and HO-1 in the wound of rats (scale bar = 50 μm, n = 5).

Based on previous studies and these findings, we hypothesize that MN@DOX+RES may stabilize Nrf2 by inhibiting Keap1-mediated ubiquitination, promote its nuclear translocation, and subsequently activate the ARE signaling pathway (Figure 11A). This pathway is expected to enhance the expression of antioxidant enzymes such as HO-1 and other cytoprotective genes, thereby restoring redox balance, alleviating oxidative and inflammatory stress, and ultimately accelerating wound healing. To verify this hypothesis, we first performed immunofluorescence staining of Nrf2 and HO-1 in RAW264.7 cells. As shown in Figure 11G, MN@DOX+RES treatment markedly enhanced the fluorescence intensity of Nrf2 and HO-1, indicating upregulation of their expression and activation of cellular antioxidant responses.

Then, we used Western blot and qRT-PCR to assess the protein expression levels of Keap1, Nrf2 and HO-1, as well as the gene expression levels of *SOD*, *CAT*, *IL-1β* and *IL-6*. The results demonstrated that following LPS stimulation, the protein expression of Keap1 was downregulated, whereas the protein expressions of Nrf2 and HO-1 were upregulated (Figure 11B). Moreover, the MN@DOX+RES treatment further potentiated this trend. In addition, MN@DOX+RES activated the Nrf2 pathway and its downstream antioxidant genes including *SOD* and *CAT* (Figure 11E-F). Compared with the control group, the LPS stimulation group showed a significant increase in the gene expression of *IL-1β* and *IL-6*, while the MN@DOX+RES treatment effectively inhibited these changes (Figure 11C-D).

To confirm whether this regulatory mechanism also occurs *in vivo*, we performed immunofluorescence staining for Nrf2 and HO-1 in infected wound tissues. As shown in Figure 11H and the semi-quantitative analysis in Figure S8, MN@DOX+RES treatment resulted in markedly elevated expression of Nrf2 and HO-1 in the wound area, consistent with *in vitro* results, indicating that this pathway is also activated within the wound microenvironment. Furthermore, Immunohistochemical analysis of IL-6 (Figure 10A, D, H) revealed that the MN@DOX+RES group had a significantly smaller area of IL-6-positive expression compared to the control and other treatment groups, indicating effective suppression of the local inflammatory response.

In conclusion, the present findings indicate that MN@DOX+RES significantly upregulates the expression of Nrf2 and its downstream antioxidant genes, such as *SOD* and *CAT*, thereby enhancing cellular antioxidant capacity. Moreover, it effectively suppresses the release of pro-inflammatory cytokines, including IL-6 and IL-1β, which in turn alleviates the inflammatory response and improves the inflammatory microenvironment in infected wounds. Collectively, these effects suggest that MN@DOX+RES holds considerable therapeutic potential for reducing oxidative stress, modulating inflammation, and mitigating tissue damage through regulation of the Keap1/Nrf2/HO-1 pathway, thus providing a promising strategy for the treatment of infected wounds.

## Conclusion

In this study, we successfully developed a bilayer drug-loaded microneedle (MN@DOX+RES), featuring a tips layer composed of ROS-responsive AHA-PBA/PVA hydrogel loaded with RES and a backing layer made of HA loaded with DOX. This bilayer microneedle MN@DOX+RES demonstrated excellent mechanical strength, efficient skin penetration, and favorable biocompatibility. The incorporation of DOX into the HA backing layer enabled its rapid release during the early phase of wound healing, thereby facilitating effective antibacterial activity. Meanwhile, the RES-loaded AHA-PBA/PVA hydrogel in the tips layer provided a sustained release into deeper subcutaneous tissues, which effectively scavenged intracellular ROS, promoted macrophage polarization toward the anti-inflammatory M2 phenotype, suppressed the expression of pro-inflammatory cytokines, and accelerated cell migration and angiogenesis. In a full-thickness infected skin wound model in rats, MN@DOX+RES significantly accelerated wound healing. Moreover, our results demonstrated that MN@DOX+RES protected cells against oxidative damage and inflammatory insults by activating the Keap1/Nrf2/HO-1 signaling pathway. This rationally designed microneedle system leveraged the distinct degradation profiles of HA and AHA-PBA/PVA hydrogels, while combining the antibacterial properties of DOX with the antioxidant and anti-inflammatory effects of RES, thereby enabling spatiotemporal sequential drug release. Overall, this work presents a promising therapeutic strategy for the effective treatment of infected soft tissue wounds.

## Supplementary Material

Supplementary figures and table.

Supplementary video.

## Figures and Tables

**Scheme 1:**
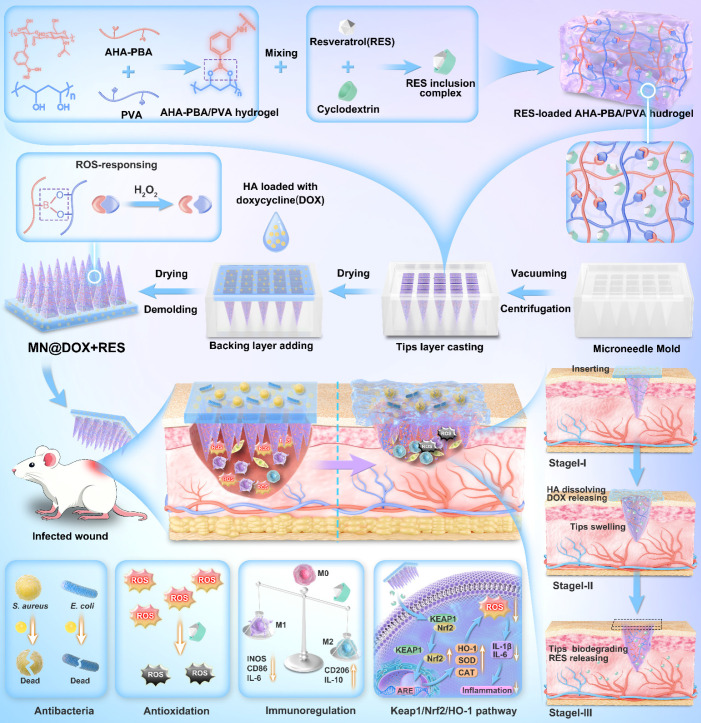
Schematic illustration of the double-layer microneedles (MN@DOX+RES) for the treatment of infected wounds.
