# Supplementary material for: ROS-Responsive Double-Layer Microneedles Enable Sequential Antibacterial and Immunomodulatory Therapy for Infected Wound Healing
Source: Theranostics. 2026 Jan 1;16(4):2052–79. doi: 10.7150/thno.122865 (PMC12680597; doi:10.7150/thno.122865)
Supplement: Supplementary file 1 — Supplementary figures and table. [file thnov16p2052s1.pdf]

ROS-Responsive Double-Layer Microneedles Enable Sequential Antibacterial and Immunomodulatory Therapy for Infected Wound Healing

Huifang Zhang[a,b,c,1], Zhongke Wang[a,b,c,1], Yujing Zhu[d], Ting Huang[a,b,c], Ziliang Xiu[e], Haozhe Huang[a.c], Hankai Li[a,c], Jing Xie[a,b,c], Haixia Huang[a,b,c], Min Liu[a,b,c], Libo Sun[f,*], Yuyan Lan[a.b.c, *], Ling Guo[a,b,c,*]

[a] Oral & Maxillofacial Reconstruction and Regeneration of Luzhou Key Laboratory, The Affiliated Stomatological Hospital, Southwest Medical University, Luzhou 646000, China

[b] Department of Prosthodontics, The Affiliated Stomatological Hospital of Southwest Medical University, Luzhou 646000, Sichuan Province, China.

[c] Institute of Stomatology, Southwest Medical University, Luzhou 646000, China

[d] Department of Stomatology, Sichuan Provincial People's Hospital, School of Medicine, University of Electronic Science and Technology of China, Chengdu 611731, China.

[e] Stomatological Hospital of Chongqing Medical University, No. 426 Songshibei Road, Yubei District, Chongqing 401147, China

[f] Department of Stomatology, Luzhou People's Hospital, Luzhou 646000, China

1 First author: Huifang Zhang and Zhongke Wang contributed equally to this work.

* Corresponding author: Libo Sun, Department of Stomatology, Luzhou People's Hospital, Luzhou 646000, China

* Corresponding author: Yuyan Lan, Oral & Maxillofacial Reconstruction and Regeneration of Luzhou Key Laboratory, The Affiliated Stomatological Hospital, Southwest Medical University, Luzhou 646000, China.

E-mail address: lyy@swmu.edu.cn

* Corresponding author: Ling Guo, Oral & Maxillofacial Reconstruction and Regeneration of Luzhou Key Laboratory, The Affiliated Stomatological Hospital, Southwest Medical University, Luzhou 646000, China.

E-mail address: glsmiling@swmu.edu.cn

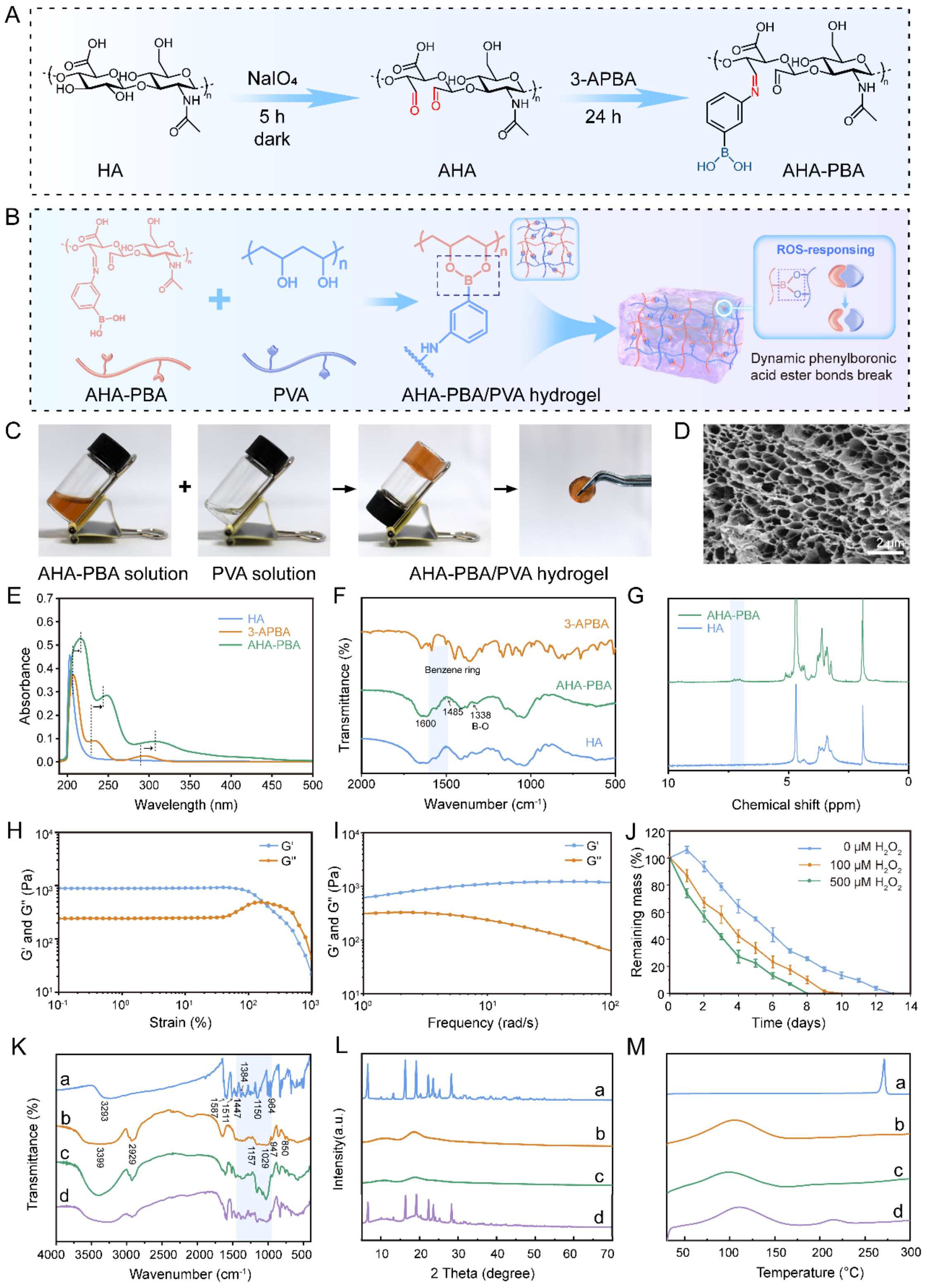

Figure S1. Successful Fabrication and Characterization of AHA-PBA/PVA Hydrogel. (A) Synthesis process of AHA-PBA. (B) Schematic of the synthesis route of AHA-PBA/PVA hydrogel. (C) Photographs of the AHA-PBA/PVA hydrogel before and after cross-linking. (D) SEM image of the freeze-dried AHA-PBA/PVA hydrogel (scale bar

= 2 μm). (E) UV-vis absorption spectra of HA, 3-APBA, AHA-PBA. (F) FTIR spectra of HA, 3-APBA and AHA-PBA. (G) ^{1}H NMR spectra of HA and AHA-PBA. (H-I) Rheological properties of AHA-PBA/PVA hydrogel: strain sweep (H) and frequency sweep (I). (J) Degradation behavior of AHA-PBA/PVA hydrogel in PBS containing 0, 100, and 500 μM H_2O_2 (n = 3). (K-M) FTIR spectra (K), XRD patterns (L), and DSC curves (M) of RES (a), HP-β-CD (b), RES inclusion complexes (c), and physical mixture (d).

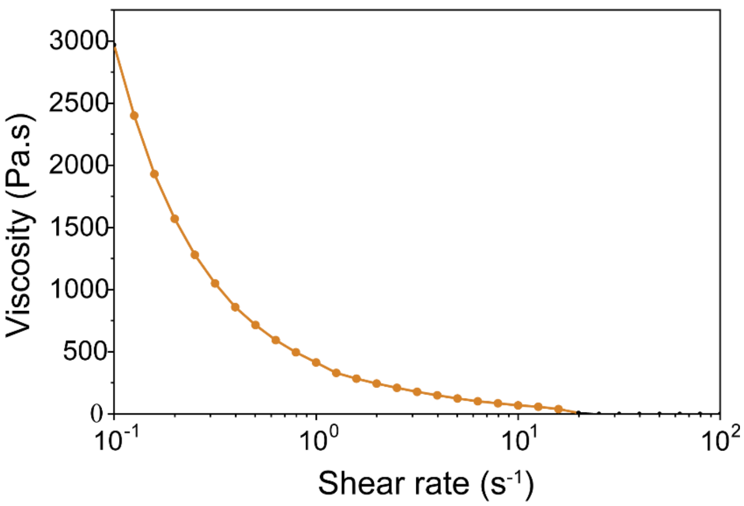

Figure S2: The viscosity of AHA-PBA/PVA hydrogels with the shear rate (0.1-100 s^{-1}).

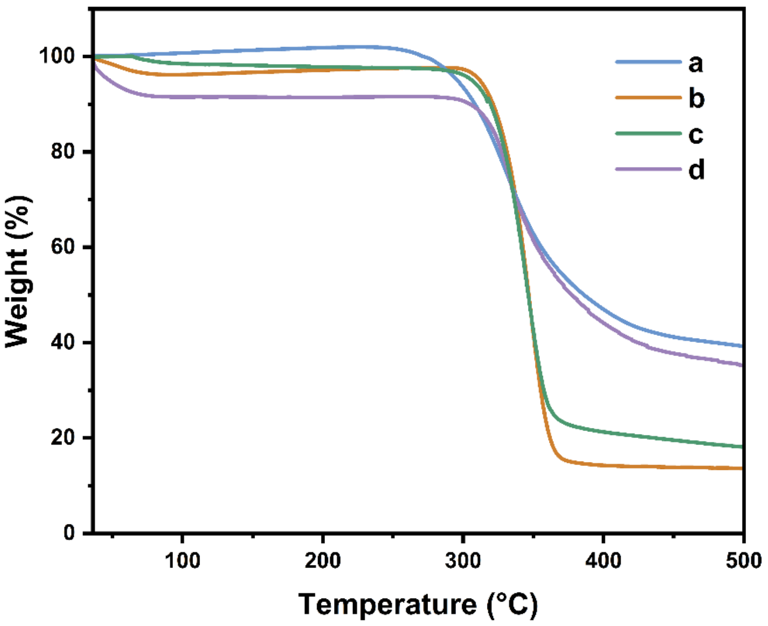

Figure S3: Thermogravimetric Characterization of RES (a), HP-β-CD (b), RES inclusion complexes (c), and physical mixture (d).

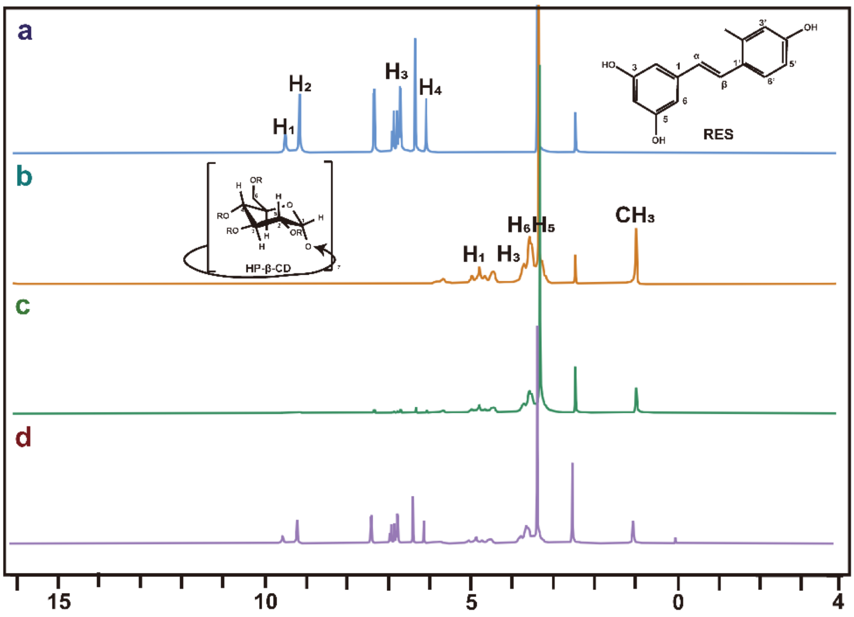

Figure S4: ^{1}H NMR characterization of RES (a), HP-β-CD (b), RES inclusion complexes (c), and physical mixture (d).

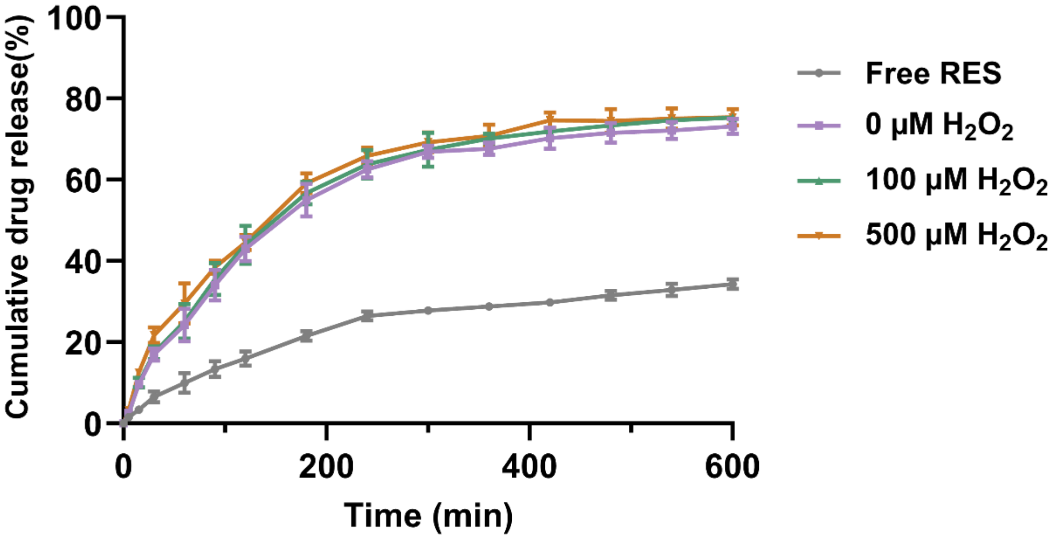

Figure S5: *In vitro* cumulative drug release profiles of free RES and RES inclusion complexes under varying concentrations of H_2O_2 (n = 3).

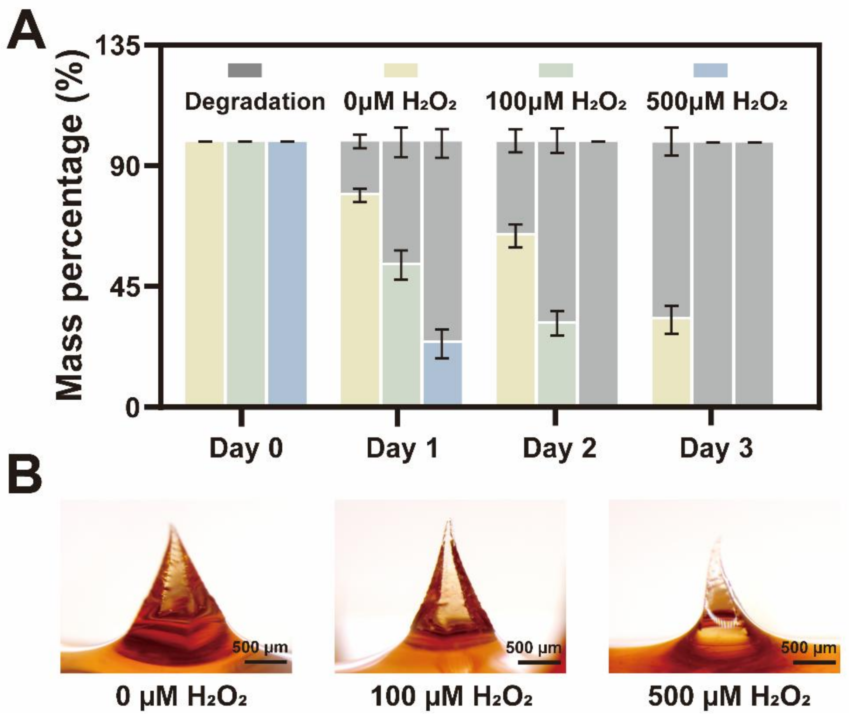

Figure S6: (A) Degradation behavior of AHA-PBA/PVA patches in PBS containing 0, 100, and 500 μM H_2O_2 (n = 3). (B) Macroscopic representative view of AHA-PBA/PVA tips after one day of degradation.

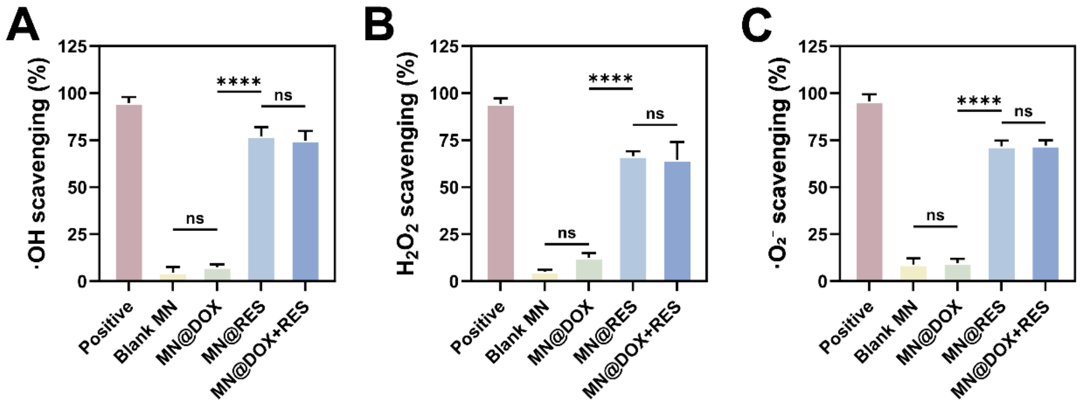

Figure S7: Quantitative Analysis of Radical Scavenging Rates for OH· (A), H_2O_2 (B), and $\cdot O_2^-$ (C). Statistical difference expression: ns, $p > 0.05$; ****$p < 0.0001$, n = 3.

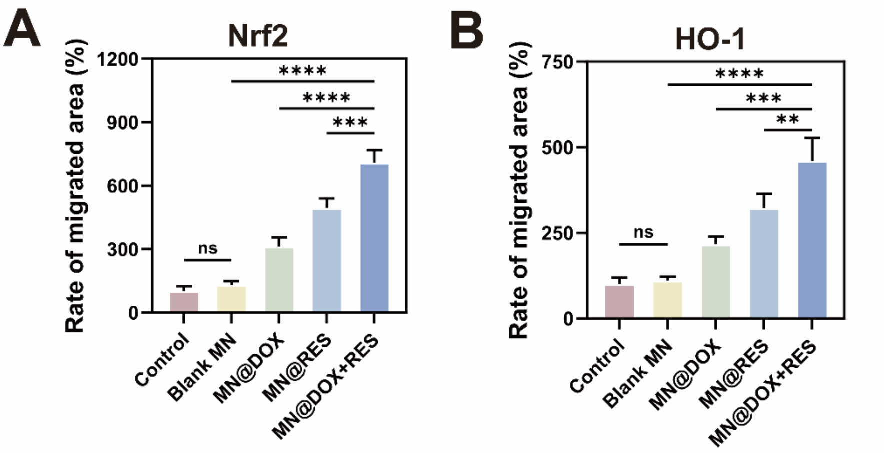

Figure S8: Semi-quantitative analysis of the relative positive areas of Nrf2 (A) and HO-1 (B). Statistical difference expression: ns, $p > 0.05$; $^{**}p < 0.01$; $^{***}p < 0.001$; $^{****}p < 0.0001$, (n = 5).

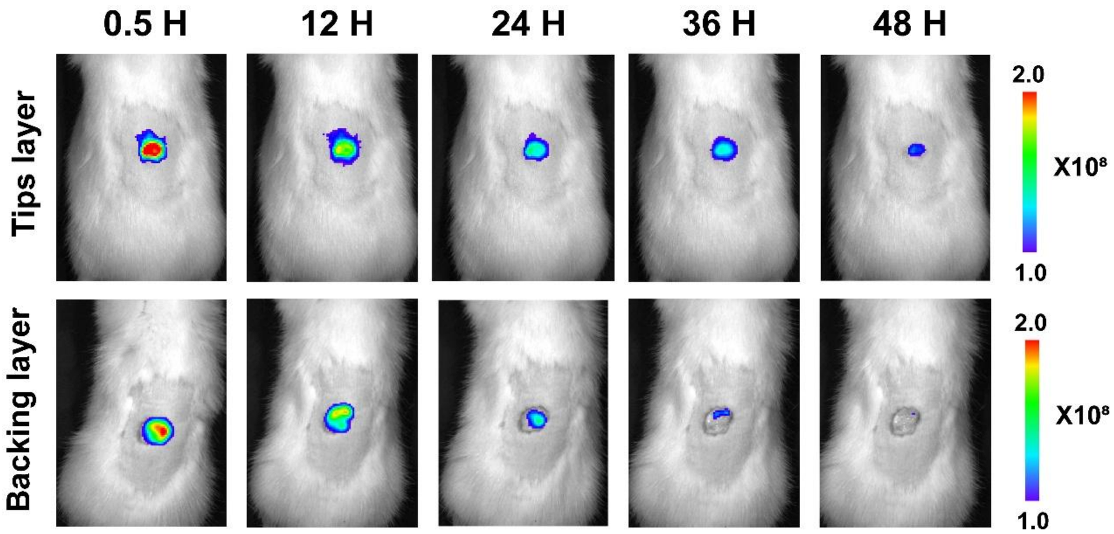

Figure S9: *In vivo* imaging in rats using MN patches loaded with fluorescent dyes.

Table S1. Primers of Keap1/Nrf2/HO-1 pathway-related genes

Gene	Primer sequence(5'-3')
β-actin	F: GTGCTATGTTGCTCTAGACTTCG
	R: ATGCCACAGGATTCCATACC
IL-6	F: TCTATACCACTTCACAAGTCGGA
	R: GAATTGCCATTGCACAACTCTTT
IL-1β	F: GAAATGCCACCTTTTGACAGTG

	R: TGGATGCTCTCATCAGGACAG
SOD-1	F: CACTTCGAGCAGAAGGCAAG
	R: CCCCATACTGATGGACGTGG
CAT	F: GGAGGCGGGAACCCAATAG
	R: GTGTGCCATCTCGTCAGTGAA
